# Unveiling the Borohydride Ion through Force-Field Development

**DOI:** 10.1021/acs.jctc.3c01020

**Published:** 2024-01-16

**Authors:** Shavkat Mamatkulov, Jakub Polák, Jamoliddin Razzokov, Lukáš Tomaník, Petr Slavíček, Joachim Dzubiella, Matej Kanduč, Jan Heyda

**Affiliations:** † Institute of Material Science of AS, Ch.Aytmatov str.2B, 100084 Tashkent, Uzbekistan; ‡ Department of Physical Chemistry, University of Chemistry and Technology, Prague, Technická 5, 16628 Prague 6, Czech Republic; § Institute of Fundamental and Applied Research, National Research University TIIAME, Kori Niyoziy 39, 100000 Tashkent, Uzbekistan; ∥ School of Engineering, Akfa University, Milliy Bog Street 264, 111221 Tashkent, Uzbekistan; ⊥ Applied Theoretical Physics–Computational Physics, Physikalisches Institut, Albert-Ludwigs-Universität Freiburg, Hermann-Herder-Str. 3, D-79104 Freiburg, Germany; # Jožef Stefan Institute, Jamova 39, 1000 Ljubljana, Slovenia

## Abstract

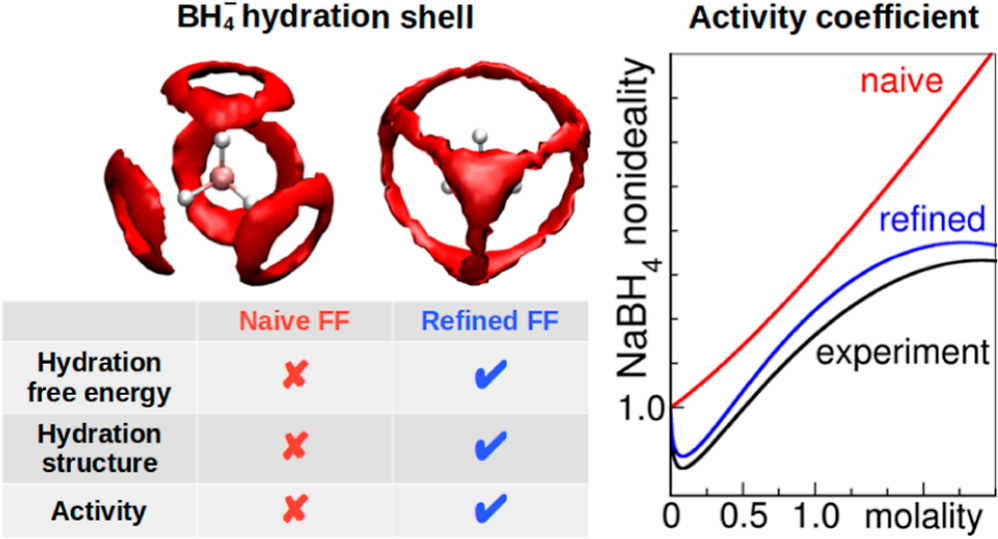

The borohydride ion, BH_4_^–^, is an essential reducing agent in many technological processes, yet its full understanding has been elusive, because of at least two significant challenges. One challenge arises from its marginal stability in aqueous solutions outside of basic pH conditions, which considerably limits the experimental thermodynamic data. The other challenge comes from its unique and atypical hydration shell, stemming from the negative excess charge on its hydrogen atoms, which complicates the accurate modeling in classical atomistic simulations. In this study, we combine experimental and computer simulation techniques to devise a classical force field for NaBH_4_ and deepen our understanding of its characteristics. We report the first measurement of the ion’s activity coefficient and extrapolate it to neutral pH conditions. Given the difficulties in directly measuring its solvation free energies, owing to its instability, we resort to quantum chemistry calculations. This combined strategy allows us to derive a set of nonpolarizable force-field parameters for the borohydride ion for classical molecular dynamics simulations. The derived force field simultaneously captures the solvation free energy, the hydration structure, as well as the activity coefficient of NaBH_4_ salt across a broad concentration range. The obtained insights into the hydration shell of the BH_4_^–^ ion are crucial for accurately modeling and understanding its interactions with other molecules, ions, materials, and interfaces.

## 1 Introduction

Sodium borohydride, NaBH_4_, is a widespread compound used for various purposes, e.g., in portable electronic devices, vehicles, electrical/thermal power plants, and fuel cells.^1^ It can store up to 10.8 mass % hydrogen, which is not flammable or explosive, and the catalytic hydrolysis of this compound provides hydrogen production under controlled conditions.^2,3^ Recent studies have predominantly focused on different catalytic systems for the hydrolysis of NaBH_4_.^4−7^ However, there is a lack of comprehensive investigations regarding borohydride in aqueous solutions. Meanwhile, it is crucial to understand the fundamentals of the solvation structure of borohydride-containing electrolytes, as it plays a critical role in electrode oxidation processes in solution.^8,9^

The proper treatment of BH_4_^–^ necessitates explicit consideration of nuclear quantum effects, owing to the low molecular weight of the boron and hydrogen atoms. Moreover, its electronic structure is complex because of the relatively weak polarization of the B–H bonds.^10^ Consequently, determining the effective partial charges for force-field simulations has become a formidable task. Gas-phase quantum chemistry calculations suggest the restrained electrostatic potential (RESP) charges of +0.525 *e* on boron and −0.175 *e* on hydrogens in the neutral BH_3_ molecule, whereas the partial charges in the BH_4_^–^ anion in a polarizable continuum are −0.110 *e* on boron and −0.2225 *e* on hydrogens. However, the situation in an explicit water environment (i.e., in an aqueous solution) is even more complicated, because of the possibility of charge transfer. In fact, BH_4_^–^ is not stable in water at pH = 7 (with decomposition kinetics on the order of seconds), where it reacts with water and decomposes into the borate anion [B(OH)_4_^–^] and gas bubbles of H_2_.^11,12^ Duffin *et al.* conducted the theoretical calculation of the hydration structure and properties of the BH_4_^–^ anion in water.^13^ The authors correlated the X-ray photoelectron spectroscopy (XPS) spectra with the excitation energies of simulated water clusters. However, their MD simulations were performed using the hybrid quantum mechanics/molecular mechanics (QM/MM) approach. The only molecule with the QM treatment was the BH_4_^–^ anion, which was modeled with the semiempirical PM6 method.

In the past, OPLS-AA force fields were developed to accurately capture the physical properties of ionic liquids, thus including parameters for less common anions such as BF_4_^–^, PF_6_^–^, or AlCl_4_^–^.^14^ The polarizable force fields were used to study structural and dynamic properties of water in aqueous solutions of structurally similar NaBF_4_.^15^ Fanfrlík *et al.*(16) studied interactions between biomolecules and carboranes, the partial charges of which were determined using the RESP method.^17^ Recently, Fang *et al.*(18,19) developed new force-field parameters of the borohydride ion by fitting *ab initio* calculations at the ωB97XD/6-311++g(3df,3pd) level of theory and compared them to X-ray experimental structural data at high NaBH_4_ concentrations. This force field has also been optimized to reproduce single-ion properties, namely, the coordination number of the BH_4_^–^ ion and the first peak of the ion–water radial distribution function (RDF). However, a plethora of studies have demonstrated that such force fields fail to produce reliable thermodynamic data at finite concentrations, and their performance can only be validated through trial and error.

In the present work, we report an optimization of a thermodynamically consistent nonpolarizable force field for the BH_4_^–^ ion based on the solvation free energy and hydration structure at infinite dilution in conjunction with the activity coefficients at finite concentrations. The ion solvation free energy is a single-ion property that describes the binding of solvation water to the ion and was calculated with the cluster-continuum approach. The activity coefficient has proven to be a sensitive indicator of ion-pairing properties and has been effectively used in the past for parameterizing ionic force fields.^20−23^ To determine the osmotic and activity coefficients of NaBH_4_ at intermediate concentrations, vapor pressure osmometry measurements were performed.

## 2 Methodology

### 2.1 Experimental Methods

#### 2.1.1 Osmotic Pressure Measurements

Osmotic pressure measurements were performed with a vapor pressure osmometer (VPO, Knauer, Berlin), which was calibrated (against NaCl standards) and equilibrated according to the instructions of the manufacturer. All measurements were performed at 37 °C (310 K) to ensure temperature stability during experiments. Prior to any experiment, the osmolality range was calibrated to the NaCl reference (400 mOsm). The osmolality of an unknown sample was then measured with respect to that reference. Osmolality (Osm), water activity (*a*_w_), and the osmotic coefficient (ϕ) are related as
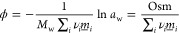
1where *M*_w_ is the water molar mass,  is the salt molality in mol·kg^–1^, and ν_*i*_ is the stoichiometric coefficient of ions in the salt molecule. In order to evaluate the activity coefficients of individual ions, we fitted the measured osmotic coefficient data with an appropriate model. We have employed the Pitzer equation, which is applicable for *n*-component electrolyte systems, with parameters for many ions available.^24^

#### 2.1.2 Application of the Pitzer Model

Application of the Pitzer model on ternary electrolyte solution followed the original work.^24^ The Pitzer equation for the osmotic coefficient of the ternary solution of a salt mixture that shares the same cation (Na^+^) is given as
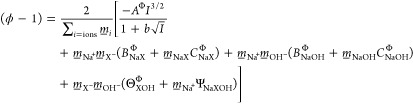
2where  is the ionic strength. The first term represents the generic Debye–Hückel contribution, while the other terms are qualitatively motivated by ion–ion interactions in a virial form, employing *B*_*i*_^Φ^, *C*_*i*_^Φ^, Θ_*i*_^Φ^, Ψ_*i*_ coefficients (the upper index Φ). We note that the cation–anion pair interaction parameter, *B*_*i*_^Φ^, is assumed to be ionic-strength-dependent *via*(24)
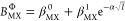
3This methodology was first applied to our newly measured VPO data (at 37 °C) of NaCl/NaOH for which the literature data and fitting parameters are available. NaCl/NaOH served as a reference for the Pitzer model on the first-time measured VPO data of the NaBH_4_/NaOH mixture.

Unfortunately, we have observed that the slow decomposition of NaBH_4_ at pH < 14, although not visually detectable, still produces enough heat to cause a misleading VPO signal of hundreds of mOsm units. Moreover, we found that repeated measurements of NaBH_4_ solutions had a detrimental impact on the thermistors of our VPO apparatus. To prevent permanent damage, we refrained from conducting additional VPO experiments and relied solely on the limited data set.

### 2.2 Hydration Free Energy of BH_4_^–^ Anions: Cluster–Continuum Approach

**Figure 1:**
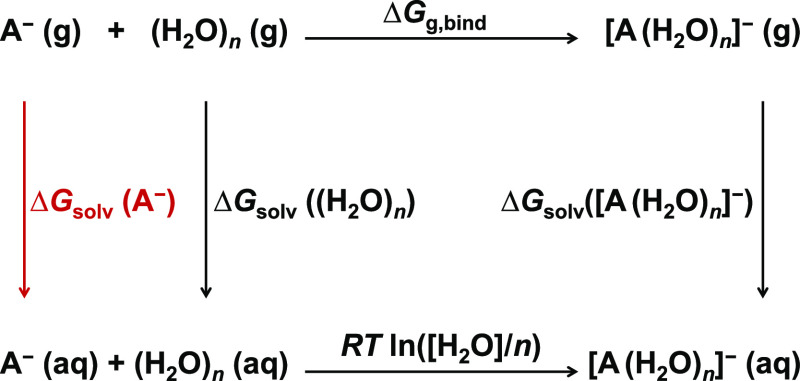
Thermodynamic cycle used within the cluster-continuum approach to calculate ionic solvation energies. A water cluster is taken from the liquid to the gas phase, where it is clustered with an ion. The resulting cluster is then solvated by an implicit solvation model. A correction to account for the different standard states is employed.

The hydration free energy, Δ*G*_solv_, is an essential property for the rational force-field development of a new ion. The magnitude of Δ*G*_solv_ is highly influenced by the geometric structure of the ion, its charge distribution, and the presence or absence of hydrogen bonds with the solvent. Here, we used the so-called cluster-continuum approach^25^ to calculate the hydration free energy of the BH_4_^–^, BF_4_^–^, and Cl^–^ anions. This method explicitly includes the closest solvent molecules, while treating the rest of the bulk solvent as a dielectric continuum. The combined approach aims to encompass both specific short-range and nonspecific long-range interactions between the solute and the solvent. Based on our previous experience,^26^ we opted to use the cluster form of this approach that is described in detail in ref (27). The computational protocol is based on a thermodynamic cycle, which is described in Figure 1.

The cycle includes the following steps: first, the gas-phase ion and a gas-phase water cluster of size *n* jointly form a mixed gas-phase cluster. Second, the formed cluster is hydrated implicitly within the dielectric continuum model. Third, correction terms are added, which are associated with the 1 mol·dm^–3^ standard state and with hydrating of the gas-phase water cluster. Finally, the hydration free energy is given as
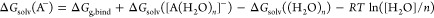
4where *R* stands for the gas constant and *T* is the absolute temperature. This approach is expected to converge with minor oscillations to a reliable value when increasing the number *n* of explicitly involved water molecules.

For each ion with a given number of explicit water molecules (*n*), we computed the terms in eq 4 as follows. We optimized structures of ions, water clusters, and mixed clusters in the gas phase to their respective energy minima, confirmed by frequency analysis. The calculations were done using the second-order Møller–Plesset (MP2) perturbation theory^28^ and the aug-cc-pVTZ basis set. Δ*G*_g,bind_ was calculated as the difference between the gas-phase free energies of the combined cluster and those of the water cluster and the ion.  and  were calculated as a difference between the electronic energy of the hydrated cluster and the gas-phase cluster. Here, hydration was described by the polarizable continuum model (PCM)^29,30^ with the Bondi atomic radii.^31^ The term *RT* ln([H_2_O]/*n*) was evaluated at a temperature of 298.15 K and the concentration of pure water of 55.34 mol·dm^–3^. All calculations were performed using the Gaussian 09 program package, revision D.01.^32^

### 2.3 Hydration Free Energy of BH_4_^–^ Anions: COSMO-RS

In addition to the cluster-continuum approach, we also employed the conductor-like screening model for real solvents (COSMO-RS) to evaluate the hydration free energies of the Cl^–^, BF_4_^–^, and BH_4_^–^ anions.^33−35^ This is a pragmatic solvent model capable of providing accurate results in a wide range of cases.^36^ First, we calculated the gas-phase and aqueous-phase properties of all three ions. The gas-phase structure was optimized using the Becke–Perdew 86 exchange–correlation functional (BP86)^37−39^ with the D3 dispersion correction,^40^ dumping according to Becke and Johnson,^41^ denoted as BP86-D3(BJ), together with the def2-TZVP^42^ basis set. Further, the optimized structure was utilized for the single-point calculation using the BP86-D3(BJ) method with the def2-TZVPD^43^ basis set. The aqueous-phase structure was optimized by employing the BP86-D3(BJ)/def2-TZVP combination and COSMO for implicit solvation. The relative permittivity value was set to ϵ_r_ = 78.39. Subsequently, the optimized structure was employed for a single-point calculation by using BP86-D3(BJ)/def2-TZVPD with the COSMO solvation and relative permittivity set to infinity. All the calculations were performed with the resolution of identity (RI) approximation with the TURBOMOLE 7.0 program.^44^ The results from the single-point calculations (both the gas phase and the aqueous phase) were then used as the input for the COSMO-RS implementation in the COSMOtherm program, version 2021, which finally provided the solvation energies of the ions.

### 2.4 *Ab Initio* MD Determination of BH_4_^–^ Hydration

*Ab initio* molecular dynamics simulations were conducted on a single BH_4_^–^ anion within a spherical cluster of 88 (quantum) water molecules with a radius ranging from 7.5 to 8.0 Å. This cluster was further surrounded by additional layers of classical water described by the TIP3P^45^ water model. The total thickness of the QM/MM hydration shell of BH_4_^–^ was 21 Å. This QM/MM onion-like cluster was embedded in the polarizable continuum model (PCM) with a water-like dielectric constant.^46^ The spherical shape of the QM/MM cavity was maintained by a harmonic restraint of *k* = 10.0 kJ·mol^–1^·Å^–2^ applied beyond a distance of 21 Å from the BH_4_^–^ anion.

The initial geometry of the spherical cluster was obtained from an equilibrated classical MD simulation (*NPT* ensemble, box side of ≈4.2 nm) performed with the force field under construction in this study. *Ab initio* simulations were carried out using the massively GPU-accelerated TeraChem simulation package^47,48^ at 300 K, employing the Nosé–Hoover thermostat and 1 fs time step. All bonds in the QM domain were flexible. The electronic wave function was calculated at the DFT-level of theory at every time step, employing the PBE0 functional with the dispersion correction of Grimme *et al.*(40)

To minimize the bias of initial conditions in the *ab initio* trajectory, we generated multiple initial configurations using two qualitatively different classical force-field simulations (corresponding to ΔΔ*G* ≈ −78 and +50 kJ·mol^–1^), which differed in the partial charge distribution in BH_4_^–^. In doing so, the initial configurations of the first hydration layer varied in their level of over- or understructuring compared to the assumed equilibrated hydration layer derived from the *ab initio* MD. In all cases, we found that 5 ps of equilibration was sufficient for the hydration shell to reach the relaxed state, and thus the water configurations sampled during the subsequent 5 ps production run were independent of the initial configuration. By conducting five independent simulations, we accumulated a total production time of 25 ps, allowing for comprehensive sampling statistics.

### 2.5 Analysis of BH_4_^–^ Hydration Structure

The division of partial charges between boron and hydrogen atoms leads to an unusual (poor) hydration of the BH_4_^–^ anion. To shed more light on this hydration shell, we have evaluated the standard one-dimensional RDF, two-dimensional angle-resolved RDF, and three-dimensional spatial distribution function. These structural functions, defined in the Supporting Information, have proven to be valuable in our previous works^49−51^ as they supplement the statistical-thermodynamic analysis of the simulation data. Typically, *ab initio* MD simulations are conducted on small systems, which lack the bulk phase, making it challenging to properly normalize their RDFs. The bulk water region incorporated in our QM/MM scheme enables normalization of RDFs to be set to 1. Simulating systems with a clusterlike shape also provides an advantage by eliminating artifacts in the charge–charge interaction across periodic images.

### 2.6 Molecular Dynamics Simulations

All classical molecular dynamics (MD) simulations were performed using the GROMACS 5.1.1 program package.^52,53^ The pair interaction potential between atoms *U*_*ij*_ was modeled through the sum of Coulomb and Lennard-Jones (LJ) interactions. Periodic boundary conditions were applied in all three directions, and the particle-mesh Ewald summation (PME) with a grid spacing of 0.12 nm in conjunction with tinfoil boundary conditions was used to handle long-ranged electrostatic forces.^54^ The simulations were carried out in the *NVT* (for equilibration) and *NPT* (for production) ensembles using the Parrinello–Rahman pressure coupling to keep the reference pressure at 1 bar^55^ and the Nosé–Hoover thermostat to keep the temperature at 300 K. The LJ interactions were truncated at 1.0 nm with a shift function for the range 0.9 nm < *r*_*ij*_ < 1.0 nm to remove jumps in the potential. We incorporated long-range corrections to account for the cutoff in LJ interactions for both energy and pressure in all our simulations. The time step used throughout the simulations was 1 fs.

We employed the SPC/E water model,^56^ which assigns partial charges of −0.8476 *e* and 0.4238 *e* to oxygen and hydrogen, respectively. The water molecule has rigid bonds of length 1.0 Å and the angle of 109.47° in between was handled by the LINCS algorithm.^57^ To calculate the single-ion solvation free energy, a borohydride ion was placed in a cubic box with a size of *L* = 2.5 nm containing 506 SPC/E water molecules. All other details of simulations are described in the Supporting Information.

### 2.7 Ion Solvation Free Energy from Ion-Pair Data

Experimental measurements of solvation free energies are limited to electroneutral ion pairs. Because of the electroneutrality, the influence of the potential jump across the air–water interface is effectively eliminated.^58^ In order to optimize the force field for an individual ion, it becomes necessary to specify the reference (counter)ion and use the difference in their solvation free energies as the optimization parameter. Following the approach described in our previous studies,^23,59,60^ we chose chloride with the Smith–Dang parameters^61^ as the reference (*vide infra*), the solvation free energy of which lies between −306^23^ and −304.2 kJ·mol^–1^.^58^

However, the data on the solvation free energy of the borohydride ion in water are very scarce, and the comparison shows severe deviations between the literature estimate^62^ and theoretical results, as we will demonstrate in the following. By employing extrathermodynamic assumptions and assuming the validity of the tetraphenylarsonium tetraphenylborate model, Marcus estimated the solvation free energies for BH_4_^–^ and Cl^–^ ions to −425 and −347 kJ·mol^–1^, respectively.^62^ These estimates yield a difference of ΔΔ*G* = Δ*G*_solv_(BH_4_^–^) – Δ*G*_solv_(Cl^–^) = −78 kJ·mol^–1^.

On the other hand, the *ab initio* values are −257 and −301 kJ·mol^–1^, respectively (*vide infra*), which leads even to a positive difference of ΔΔ*G* = 44 kJ·mol^–1^ (≈50 kJ·mol^–1^). Yet, the upper bound is to combine the *ab initio* values for qualitatively similar ions BH_4_^–^ (−257 kJ·mol^–1^) and BF_4_^–^ (−230 kJ·mol^–1^) with reliable experimental values of Cl^–^ (−347 kJ·mol^–1^) and BF_4_^–^ (−200 kJ·mol^–1^), giving us ΔΔ*G* = 120 kJ·mol^–1^.

Because of the significant discrepancy between the literature estimate^62,63^ and theoretical data, we are unable to place confidence in any specific value. Consequently, we have decided to leave this parameter open for further investigation. We will consider several possibilities for ΔΔ*G*, namely −78, 50, and 100 kJ·mol^–1^.

### 2.8 Kirkwood–Buff Integration

We used the Kirkwood–Buff theory to obtain the activity derivatives of the salt solutions from MD simulations, as was done before in similar studies.^23^ The required RDFs, *g*(*r*), were obtained from separate simulations in the *NPT* ensemble. The simulation box (a cubic box with side lengths of *L* = 4 nm) contained 20 and 40 BH_4_^–^/Na^+^ ion pairs together with 2130 and 2072 SPC/E water molecules, which yields solutions with concentrations of 0.5 and 1.0 mol·kg^–1^, respectively. For evaluating the RDFs at higher concentrations, 2 and 3 mol·kg^–1^, we used a cubic box *L* = 6 nm in size, containing about 5000 water molecules and 200 and 300 Na^+^/BH_4_^–^ pairs, respectively. The simulations were performed with a 2 fs time step. To gather sufficient statistics, the particle trajectories were stored every 0.2 ps throughout a total simulation time of 100 ns.

The logarithmic derivative of the activity with respect to the density ρ = *N*_+_/*V* = *N*_–_/*V* equals the following combination of Kirkwood–Buff integrals
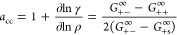
5The subscripts +, −, and s denote cation, anion, and solvent, respectively, and γ stands for the mean activity coefficient of anions and cations. The Kirkwood–Buff integrals in eq 5 were calculated as
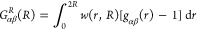
6where *R* are the radii of the probe-spheres around particle α from which extrapolation (1/*R* → 0) of the thermodynamic Kirkwood–Buff integral (*G*_αβ_) is evaluated,^64^*g*_αβ_(*r*) is the RDF between species α and β, and the geometrical weight function is given by^65^
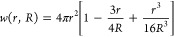
7To achieve accurate results, it is crucial to properly normalize the RDF due to finite-size effects. To this end, we multiply the evaluated RDF from a simulation trajectory, *g*_αβ,sim_(*r*), by a correction factor, *f*, so that the corrected RDF, *g*_αβ_(*r*) = *f*·*g*_αβ,sim_(*r*), reaches unity over large distances. Additional details on the Kirkwood–Buff theory can be found elsewhere.^23^

## 3 Results and Discussion

### 3.1 VPO Measurements

Before delving into the force field construction, we gain valuable thermodynamic insight into NaBH_4_ solutions using vapor pressure osmometry (VPO) measurements. In this way, we were able to determine the osmotic coefficient at intermediate concentrations, providing crucial information about the behavior of NaBH_4_ in solution. So far, the only activity data were available near saturation (>10 mol·kg^–1^) from the early work of Stockmayer *et al.*(66)

VPO measurements, a routine procedure in most cases, become very challenging when it comes to NaBH_4_ because of its instability in pure water at a neutral pH. To overcome this issue, we have employed the following extrapolation scheme. We conducted a series of VPO measurements using mixed salt/NaOH solutions, starting with high pH and gradually decreasing it. The results were then extrapolated to estimate the osmotic coefficient at pH = 7, corresponding to a solution without NaOH.

**Figure 2:**
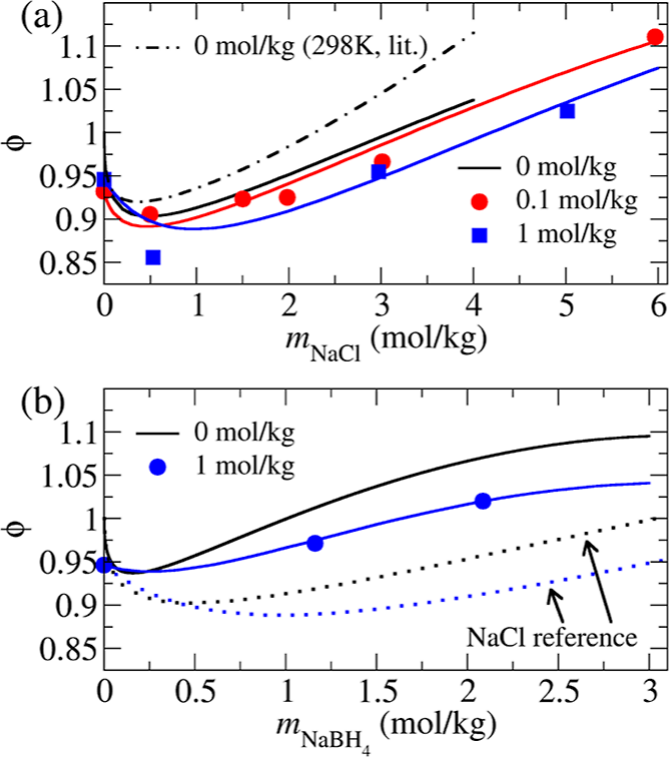
Extrapolation scheme for estimating the osmotic coefficient ϕ of salt at pH = 7 from a series of our VPO measurements of the salt at basic pH (≈13–14) in the presence of NaOH using the Pitzer equation for mixed electrolytes (eq 2). (a) NaCl/NaOH data points (Table S1) of the ternary mixture are present at different NaOH concentrations (see the legend). Fits of eq 2 are presented as solid lines (fit parameters are summarized in Table 1). Extrapolation to pH = 7 (i.e., NaCl solution at ) is represented by the solid black line. The osmotic coefficient for NaCl/water at 298 K, calculated using the Pitzer equation,^24^ is depicted by the black dash-dotted line as a reference. (b) NaBH_4_/NaOH (1 mol·kg^–1^) ternary mixture evaluated by the same protocol as for NaCl/NaOH in panel (a). Data points (Table 2) were fitted (blue solid line, parameters in Table 3) and extrapolated to a binary NaBH_4_/water mixture (black solid line). For reference, the NaCl/NaOH systems under the same conditions are shown (dotted lines).

We first applied the method to published VPO data^24^ (at 37 °C) of NaCl/NaOH for which fitting parameters were available. Data on ternary NaCl/NaOH mixed solutions at several pH levels (13–14) are summarized in Table S1. The application and quality of the extrapolation scheme perform very well, as illustrated in Figure 2a. The readjusted coefficients of the Pitzer equation that represent osmotic coefficients at 310 K (computed with eq 1) are indicated by the asterisk (*) and summarized in Table 1. The coefficients served as the reference for the Pitzer model on first-time measured VPO data of the NaBH_4_/NaOH mixture. Mind that in aqueous solutions, NaBH_4_ decomposes into hydrogen gas and sodium borate *via* a reaction catalyzed by H^+^ cations present in the solution. However, this reaction can be inhibited only at extremely high pH levels (≈14). Thus, only the experimental data at  = 1.0 mol·kg^–1^ were used in the analysis. The raw experimental data are presented in Table 2, in which the water activity and osmotic coefficient were evaluated according to eq 1.

**Table 1:** Fit Parameters for NaCl/NaOH, When Molality Concentration Scale Is Used^a^

*A*^Φ^	Θ_ClOH_	Ψ_NaClOH_	*C*_NaCl_^Φ^	*C*_NaOH_^Φ^	β_NaCl_^0^	β_NaCl_^1^	β_NaOH_^0^	β_NaOH_^1^	α
0.393991	–0.2018*	0.006898*	–0.00346*	0.0044	0.080155*	0.116695*	0.0864	0.253	2

a Asterisk (*) indicates the parameters determined by fitting.

**Table 2:** Osmolality of Ternary NaBH_4_/NaOH Mixed Solutions as Determined in VPO Measurements at 310 K, Which Serves for Determining the Remaining Free Parameters of the Pitzer Model (See Table 3)

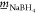	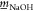	Osm (mmol·kg^–1^)	ln *a*_w_	ϕ
1.1434	0.9999	4096	–0.07424	0.962
1.1807	0.9999	4245	–0.07694	0.980
2.0854	0.9999	6249	–0.11327	1.020

The data collected at  = 0.1 mol·kg^–1^ (pH ≈ 13) was disregarded since our experiments revealed that the heat associated with the NaBH_4_ decomposition interfered with the VPO signal. Even though the decomposition process (e.g., formation of H_2_ bubbles) was not visually apparent during the experiment, the VPO instrument detected an abnormal signal of hundreds of mOsm units.

We utilized the protocol approved on the NaCl/NaOH system to derive BH_4_^–^-related coefficients. For this, we applied the least-squares method optimization to the data presented in Table 2, as illustrated in Figure 2b. Due to the lack of data at different pH levels, we did not attempt to determine the two- and three-body coefficients,  and , respectively. Instead, we assumed them to be equivalent to those in the NaCl/NaOH system, which renders them salt-nonspecific. The Pitzer parameters for the NaBH_4_/NaOH mixture are summarized in Table 3.

**Table 3:** Fit Parameters for NaBH_4_/NaOH, When Molality Concentration Scale Is Used^a^

*A*^Φ^	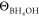	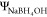	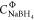	*C*_NaOH_^Φ^	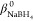	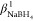	β_NaOH_^0^	β_NaOH_^1^	α
0.393991	–0.2018	0.006898	–0.02448*	0.0044	0.171824*	0.23078*	0.0864	0.253	2

a Asterisks (*) indicate the parameters that were readjusted to fit the experimental data of the NaBH_4_/NaOH mixture as present in Figure 2.

Based on the osmotic coefficient parameters for the binary NaBH_4_/water solution (i.e., at pH = 7), we have evaluated the activity coefficient (γ_NaBH4_) and its derivative
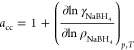
8see Figure S1 in the Supporting Information. This thermodynamic parameter (*vide infra*) will serve as a quantitative benchmark for the NaBH_4_ force field at intermediate and high concentrations.

### 3.2 QM-Calculation of Hydration Free Energy

To calculate the hydration free energy of the BH_4_^–^ anion, as well as two reference anions (Cl^–^ and BF_4_^–^), we employed the cluster-continuum approach (CCA)^27^ with an increasing number of explicit QM water molecules (ranging from 0 to 3, see Table S4 in the Supporting Information). As already stated above, the free energy change for the alchemical transformation Cl^–^ → BH_4_^–^ based on the literature data^67^ (column 3 in Table 4) is ΔΔ*G* = −78 kJ·mol^–1^. However, the CCA values (column 1 in Table 4) provide a qualitatively different picture: They yield a positive value of ΔΔ*G* = 44 kJ·mol^–1^. Another estimate for ΔΔ*G* is to make use of the known experimental difference between Cl^–^ and BF_4_^–^, which is robust, and correct it with the difference in solvation energies between BH_4_^–^ and BF_4_^–^ from the theoretical calculation. Given the similar charge distribution and hydration structure of these two anions, the difference in their hydration free energy is expected to be more reliable, namely
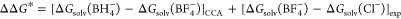
9giving 120 kJ·mol^–1^. With this bound, the theoretical estimate spans across ΔΔ*G* = 44–120 kJ·mol^–1^ (Table 4).

**Table 4:** Hydration Free Energy of Borohydride and Other Reference Anions (Cl^–^ and BF_4_^–^) as Calculated with the Cluster-Continuum (CCA, Average 1–3 Water Molecules, See Table S4), COSMO-RS Approaches, and Estimated in the Literature^62,63,67^^a^

	CCA	COSMO-RS	Lit. estimate^62^
BF_4_^–^	–230	–257	–200
Cl^–^	–301	–304	–347
BH_4_^–^	–257	–254	–425
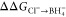	–78	44	50
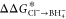	n.a.	120	150

a Energies are given in kJ·mol^–1^. The lower bound of  is calculated directly as ΔΔ*G* = Δ*G*_solv_(BH_4_^–^) – Δ*G*_solv_(Cl^–^), while the upper bound employs the known difference^62^ between Cl^–^ and BF_4_^–^, calculated *via*eq 9.

The significant discrepancy of at least 122 kJ·mol^–1^ between the literature estimate^62,63^ and calculated values suggests that the hydration structure of BH_4_^–^ is very different from those of small halides like Cl^–^ (see Figure S6 in the Supporting Information), which coordinates water molecules well. This notion is reinforced by the optimized geometries of hydrated anions obtained in QM calculations (see Figure S6 in the Supporting Information). The observations indicate that BH_4_^–^ shares a similar hydration structure and polarity with poorly hydrated anions, such as BF_4_^–^. These findings have crucial implications for force-field development since they necessitate a substantial redistribution of partial charges in the anion, ultimately reducing its polarity and propensity for hydrogen bonding.

Overall, our findings cast doubt on the reliability of the estimated value for Δ*G*_solv_ for BH_4_^–^.^62,63^ To further strengthen the reliability of the CCA results, we conducted COSMO-RS calculations, which are conceptually different but yielded quantitatively similar outcomes, with ΔΔ*G* = 50–150 kJ·mol^–1^ (see Table 4). We may hypothesize that the large discrepancy (by at least 122 kJ·mol^–1^) from the literature estimate arises from the decomposition characteristics of borohydride under neutral conditions (pH = 7).

### 3.3 Constructing the Force Field

Because of the tetrahedral structure of the BH_4_^–^ anion, the boron atom is fully surrounded and encapsulated by hydrogen atoms. As a result, boron size has minimal impact on the solvation of the ion in water (which we demonstrate in Figure S8 in Supporting Information). Therefore, in the force field parametrization, we focus on fine-tuning only both Lennard-Jones (LJ) parameters of the hydrogen atoms, namely, σ_HB_ and ε_HB_. For the LJ parameters of boron and the partial charges, we adopt the values from the force field developed by Fang *et al.*,^18^ which are σ_B_ = 0.348 nm, ε_B_ = 0.4 kJ·mol^–1^, and *q*_B_ = 0.108 *e*. The reason to adopt the partial charges by Fang *et al.* is that we obtained a very similar result using the CHELPG method.^68^

**Figure 3:**
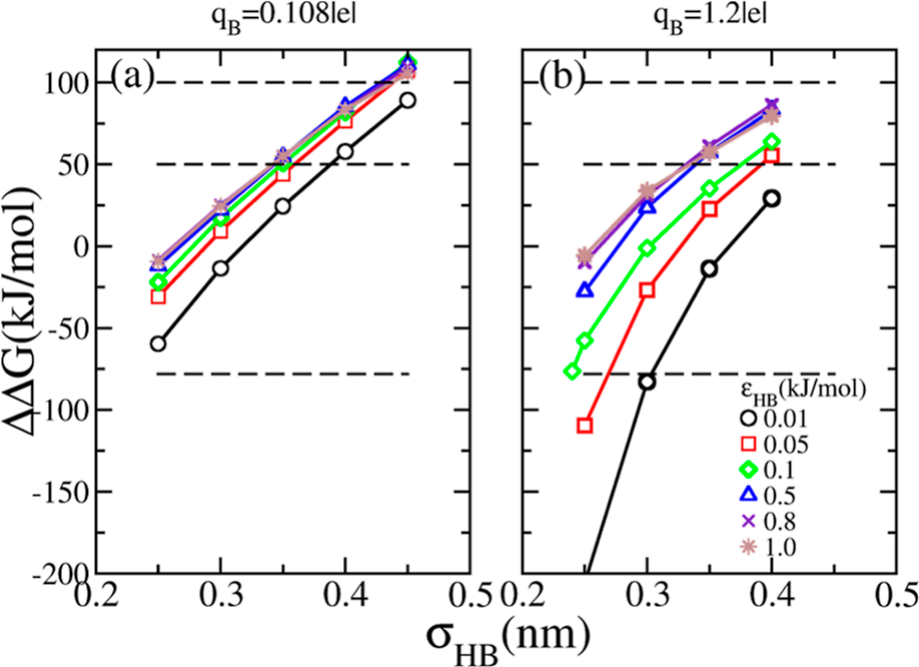
Solvation free energy difference ΔΔ*G* between the borohydride and chloride ions as a function of σ_HB_. The LJ parameters of boron are fixed to σ_B_ = 0.348 nm and ε_B_ = 0.4 kJ·mol^–1^. Simulation results are shown by symbols for different LJ parameters ε_HB_ and partial charges *q*_B_. The horizontal (black dashed) lines represent the literature estimate (−78 kJ·mol^–1^) as well as our estimates based on the *ab initio* results (50 and 100 kJ·mol^–1^).

We begin our analysis by examining how the solvation free energy difference ΔΔ*G* between borohydride and chloride ions depends on σ_HB_ and ε_HB_, which we plot in Figure 3. The horizontal dashed lines in the plot denote our propositions for the free energy of ΔΔ*G* = 50 and 100 kJ·mol^–1^, along with the qualitatively different experimental estimate ΔΔ*G* = −78 kJ·mol^–1^^67^ (see discussion of Table 4). From Figure 3a it is apparent that the curves for the used partial charges cannot reach the experimental value within a meaningful interval of σ_HB_. The experimental value can be reached only by using much larger partial charges (*q*_B_ > 0.8 *e*). Therefore, in an effort to explore a potential model that reproduces this value, we also investigate the parameterization with partial charges of *q*_B_ = 1.2 *e* and *q*_H_ = −0.55 *e*, as shown in Figure 3b.

**Figure 4:**
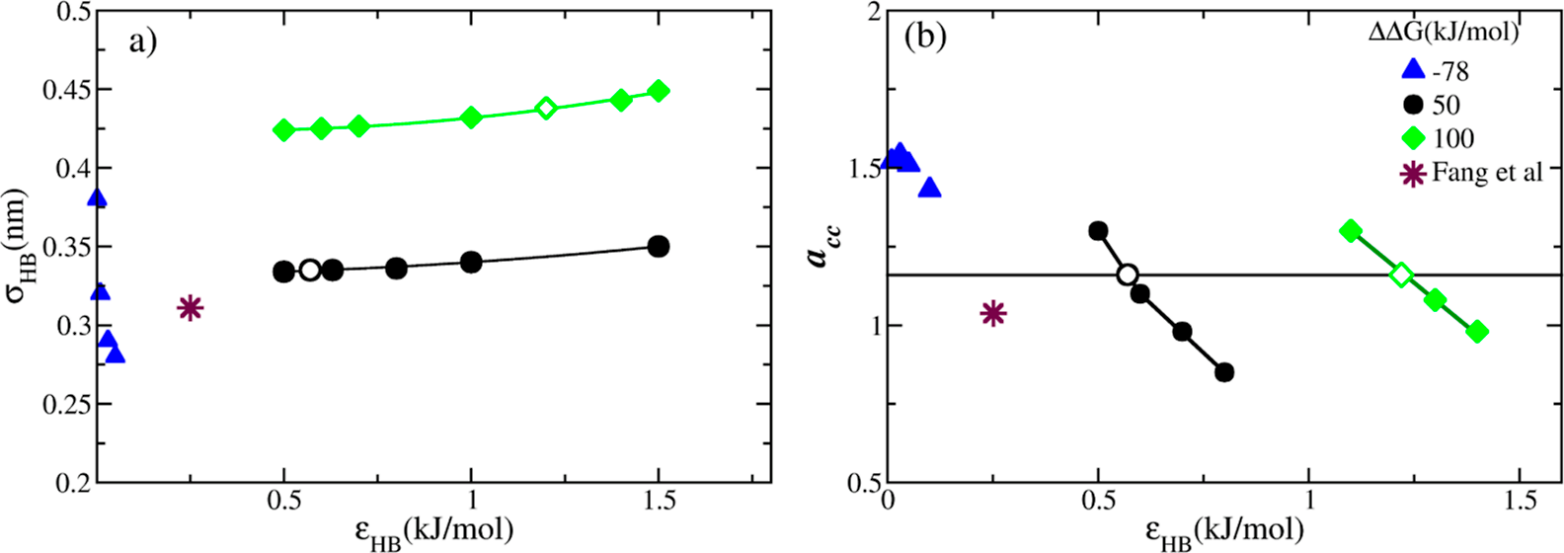
(a) Solvation free energy isolines of the BH_4_^–^ ion in the σ_HB_–ε_HB_ space, extracted from Figure 3a with *q*_B_ = 0.108 *e*. The solid lines are fits of parabolic functions. (b) Activity coefficient derivatives of a 2 mol·kg^–1^ NaBH_4_ electrolyte as a function of ε_HB_. The matches between experimental (horizontal solid line *a*_cc_ = 1.18) and simulated values are listed in Table 5. The star symbol represents the value obtained using the force field by Fang *et al.*(19)

In the next step, we identify the combinations of LJ parameters that accurately reproduce various solvation free energies, as depicted in Figure 4a. Subsequently, in Figure 4b, we compute the activity derivatives *a*_cc_ of a 2 mol·dm^–3^ NaBH_4_ solution (using eq 5) as a function of ε_HB_. The experimental value (*a*_cc_ = 1.18) is also included for comparison as a solid line. For each set of ΔΔ*G*, we determine the combination of LJ parameters that matches the activity derivative of NaBH_4_ (indicated by open symbols). This gives us four sets of parameterizations, listed in Table 5, which serve as potential candidates for the final force field selection.

**Table 5:** Candidates for the Final Force Field: Lennard-Jones Parameters and Partial Charges That Reproduce Various Solvation Free Energies ΔΔ*G*^a^

ΔΔ*G* (kJ·mol^–1^)	sites	σ_*i*_ (nm)	ε_*i*_ (kJ·mol^–1^)	*q* (*e*)
–78	B	0.348	0.4	1.2
	H_B_	0.27	0.05	–0.55
50	B	0.348	0.4	0.108
	H_B_	0.34	0.57	–0.277
100	B	0.348	0.4	0.108
	H_B_	0.43	1.2	–0.277

a Atomic partial charges for ΔΔ*G* = 50 and 100 kJ·mol^–1^ are taken from Fang *et al.*(19)

**Figure 5:**
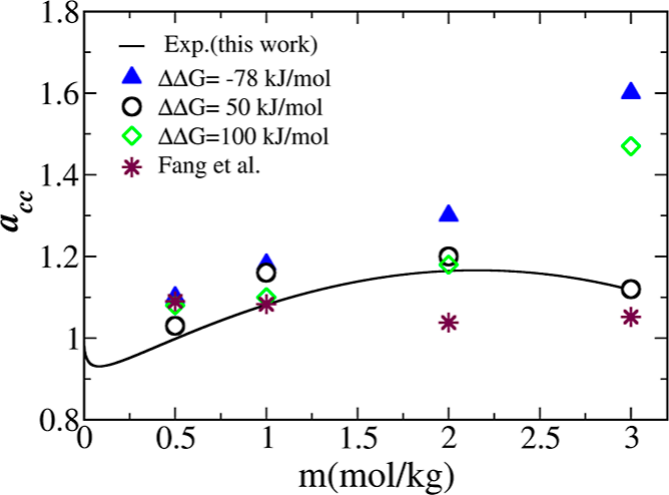
Comparison of NaBH_4_ activity coefficient derivative as a function of concentration using the force field parameterizations in Table 5 as well as the force field by Fang *et al.*(19) Activity derivatives are calculated for LJ parameters corresponding to different ΔΔ*G* values. The LJ parameters for ΔΔ*G* = 50 kJ·mol^–1^, σ_HB_ = 0.34 nm, and ε_HB_ = 0.57 nm (open circles) show a good match with the experimental data (black curve).

To scrutinize the accuracy of the identified parametrizations, we further examine their ability to reproduce the activity derivative of NaBH_4_ at varying concentrations. This analysis is shown in Figure 5, in which the simulation results are represented by symbols, whereas the experimental value obtained through eq 5 (Section 3.1, and Figure S1 in the Supporting Information) is depicted by a solid line. It can be seen that the set of LJ parameters for ΔΔ*G* = 50 kJ·mol^–1^ (open circles) gives the best match to the experimental data over the entire concentration range. The other sets fail at higher concentrations (*m* > 2 mol·kg^–1^), along with the force field by Fang *et al.*(19) (maroon stars). Too small values of *a*_cc_ indicate too strong ion pairing and the other way around for too high values of *a*_cc_.

The optimization of LJ parameters has relied solely on reproducing thermodynamic quantities, the activity coefficient (derived experimentally), and the solvation free energy (obtained from *ab initio*). While this approach has been sufficient to determine the force field, it is highly beneficial to extend the analysis to encompass microscopic structural properties derived from *ab initio* simulations, specifically the RDFs and coordination numbers. Doing so will not only validate the force field but will also provide further insights and a more comprehensive understanding of the borohydride ion.

**Figure 6:**
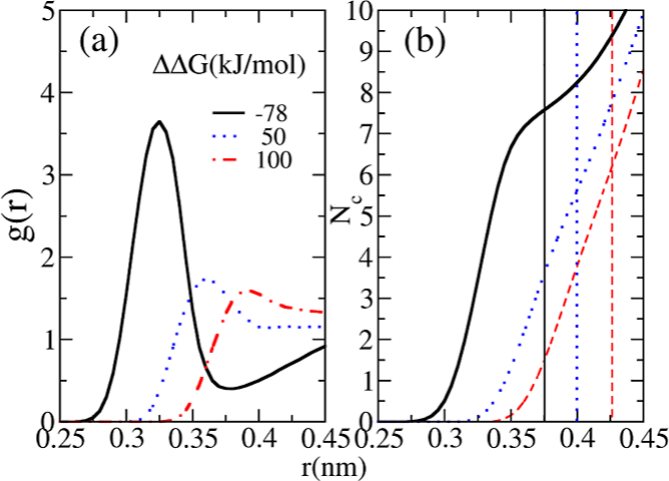
(a) Radial distribution function of the boron and oxygen atoms of water (B–O_w_) in infinite dilution using different parameterizations in Table 5. (b) Coordination number of BH_4_^–^ ions obtained by integrating the RDFs from panel (a). The vertical lines correspond to the minima following the primary peak in RDFs, denoting the end of the first hydration shell.

In Figure 6a, we compare RDFs between boron and water oxygen atoms stemming from the parametrizations listed in Table 5. By integrating the RDFs, we obtain the cumulative number of water molecules surrounding the BH_4_^–^ ion, as shown in Figure 6b. The minimum following the main peak of an RDF (designated also by vertical lines at 0.375, 0.4, and 0.425 nm) denotes the end of the first hydration shell, which can be used to estimate the coordination number of the borohydride ion. The three parameterizations yield the coordination numbers of *N*_c_ = 7.5, 5.6, and 6.2 for ΔΔ*G* = −78, 50, and 100 kJ·mol^–1^, respectively. The hydration number of *N*_c_ = 5.6 obtained for ΔΔ*G* = 50 kJ·mol^–1^ is consistent with the *ab initio* result of 5.8 ± 1.4 reported by Fang *et al.* at 0.973 mol·dm^–3^ and pH ≈ 14.^19^ The parameter set that reproduces ΔΔ*G* = −78 kJ·mol^–1^ overestimates the coordination number by a large margin, which further undermines the reliability of the solvation free energy measurement. In conclusion, the analysis of coordination numbers obtained by different parametrizations (Table 5) further reinforces the quality of the set corresponding to ΔΔ*G* = 50 kJ·mol^–1^. Consequently, we choose this particular set as the definitive force field. In Table 6, we summarize the final force field for BH_4_^–^, together with the parameters for Na^+^ and SPC/E water.

**Table 6:** Final Force Field of the BH_4_^–^ Ion Obtained in This Work along with Water and Sodium Ion Parameters^a^

sites	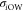 (nm)	(kJ·mol^–1^)	σ_*i*_ (nm)	ε_*i*_ (kJ·mol^–1^)	*q* (*e*)	refs
B	0.34	0.51	0.358	0.4	0.108	(19)
H(BH_4_)	0.31	0.4	0.31	0.25	–0.277	(19)
B	0.332	0.51	0.348	0.4	0.108	this work
H(BH_4_)	0.33	0.61	0.34	0.57	–0.277	this work
Na^+^	0.287	0.521	0.258	0.42	1	(61)
Cl^–^	0.378	0.521	0.440	0.42	–1	(61)
O of water	0.3166	0.65	0.3166	0.65	–0.8476	(56)
H of water	0.000	0.00	0.000	0.00	0.4238	(56)

bond type	interaction function	*r*_0_ (nm)	*k*_b_ (kJ·mol^–1^·nm^–4^)	refs
B–H	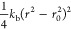	0.124	3.7656 × 10^5^	(70)
O–H of water	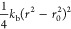	0.10	3.4500 × 10^5^	(56)

angle types	interaction	θ_0_ (degrees)	**k*_θ_* (kJ·mol^–1^)	refs
∠H–B–H	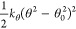	109.5	520.0	(70)
∠H–O–H	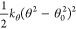	109.47	383.0	(56)

a The Lorentz–Berthelot mixing rules are used for σ_*ij*_ and ε_*ij*_.

**Figure 7:**
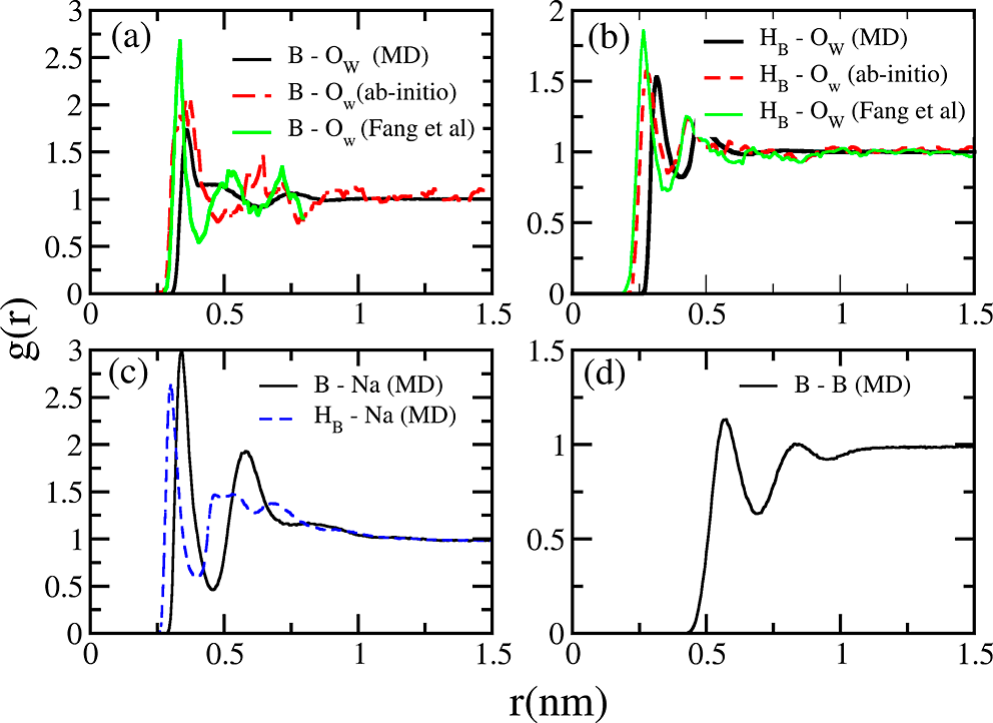
Radial distribution function of BH_4_^–^, Na^+^, and water obtained from the final force field (Table 6) and *ab initio* simulations: (a) Boron and water oxygen from MD (solid black line), *ab initio* from this work (red dashed line) and *ab initio* from Fang *et al.*(19) (green line). (b) Borohydrate hydrogen and water oxygen from MD (black solid line) and from *ab initio* (red dashed line). (c) Sodium–boron (black line) and sodium–hydrogen of BH_4_^–^ (blue dashed line) MD results. (d) Boron–boron from MD simulations (black line). The RDFs were computed from simulations of 1 M NaBH_4_ solutions.

To conduct a conclusive evaluation of the new model, we thoroughly analyze the consistency of its RDFs with those obtained from *ab initio* simulations. In Figure 7a,b, we compare RDFs of BH_4_^–^ and water at infinite dilution (solid lines) to those from *ab initio* simulations (dashed lines). The peak positions of boron and water oxygen from force-field simulations (Figure 7a) closely match those from *ab initio* simulations, with values of 0.36 and 0.32 nm, respectively. The closest distance between the borohydride hydrogen atoms and water oxygen atoms (Figure 7b) as determined from force-field simulations is 0.31 nm (solid line), which is slightly above the *ab initio* value of 0.26 nm (dashed line).

The activity coefficient (relevant at finite concentrations), which was used in the force-field optimization, is primarily governed by ion–ion interactions (eq 5). Simulation results at 1 mol·kg^–1^ NaBH_4_ indicate that Na^+^ and BH_4_^–^ preferably form a contact ion pair with a Na–B distance of 0.322 nm (Figure 7c). The boron–boron RDF shows a weak and broad peak at 0.6 nm (Figure 7d), which suggests the formation of both solvent- and sodium-separated borohydride pairs.

**Figure 8:**
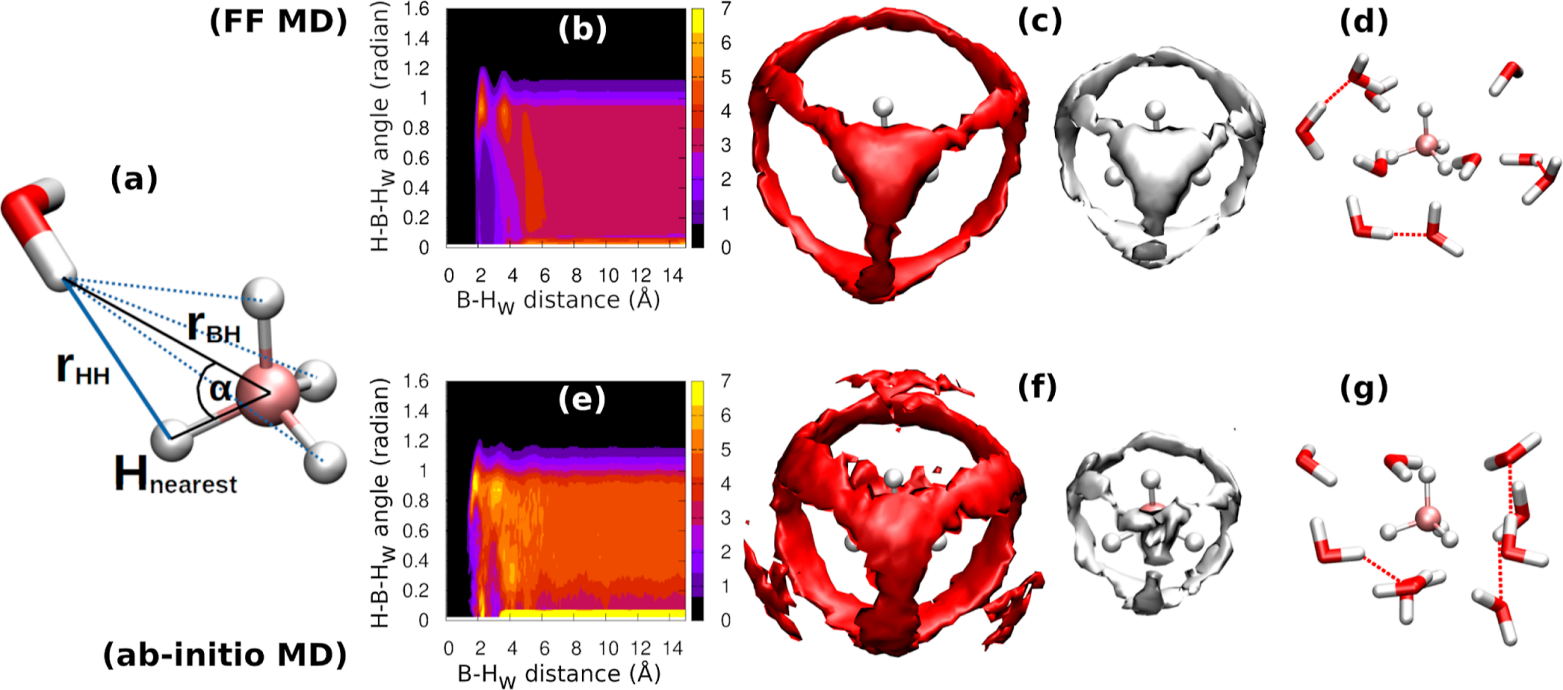
Insight into the local hydration of BH_4_^–^ described by our novel force field (top row) and compared to the *ab initio* (PBE0) MD results (bottom row, see the Supporting Information for details). (a) Definition of distances and angles for the calculation of *g*(*r*, α). The distances to water hydrogen atoms are measured from the boron atom. The angle α is defined between the closest boron hydrogen, boron, and water hydrogen. (b,e) Angle-resolved probability distribution of H_w_–H_B_, *g*(*r*, α), employing the definition of α-angle and distance from panel (a). (c,f) Spatial distribution of oxygen (red clouds, isocontour at 3× the bulk density) and hydrogen (white clouds, 2× the bulk density) atoms of water in the proximity of borohydride. (d,g) Example geometry of a rather disordered hydration layer of a borohydride, where we have highlighted preserved H-bonds among hydration water molecules. Contrasting results, stemming from an overly polar force field of BH_4_^–^ (ΔΔ*G* = −78 kJ·mol^–1^), are provided in the Supporting Information (Figures S2–S5).

Finally, in Figure 8, we present two-dimensional angle and three-dimensional spatially resolved details of the borohydride hydration. Employing the angle definition in Figure 8a, we observe that water hydrogen atoms (H_w_) exhibit an isotropic (random) orientation relative to the B–H bonds of borohydride (classical MD in Figure 8b, *ab initio* MD in Figure 8e), indicating the absence of a directional preference for the H_B_–H_w_ bond. This is further supported by the spatial distribution function (classical MD in Figure 8c, *ab initio* MD in Figure 8f), where the densities of water oxygen and hydrogen atoms (depicted as red and white clouds, respectively) are scattered around the borohydride.

The consistency between the force-field and *ab initio* simulations is further reinforced by the resemblance in the water structure of the first hydration shell (random snapshot) of borohydride (compare Figure 8d and 8g). We observe that the H-bond network of water molecules within the first hydration layer is preserved, effectively encapsulating the approximately spherical BH_4_^–^ anion. It is worth noting that because of small partial charges on the borohydride hydrogen atoms, the solvation behavior is qualitatively similar to that of methane.^69^

We conclude that our force field shows quantitative agreement with *ab initio* MD simulations for the hydration structure of the BH_4_^–^ anion. In contrast, the highly polar force field (reproducing ΔΔ*G* = −78 kJ·mol^–1^, as presented in Figures S2–S5) exhibits significant deviations and inaccuracies. In summary, the new parametrization of the borohydride ion exhibits a remarkable level of consistency when compared to experimental data and *ab initio* results.

## 4 Conclusions

In this work, we report an optimization of a nonpolarizable atomistic force field for BH_4_^–^ based on its solvation free energy in water and the thermodynamic activities of Na^+^/BH_4_^–^ electrolyte solutions. For the first time, the osmotic pressure coefficients of the NaBH_4_/NaOH system were measured by using the vapor osmometric pressure method. Additionally, the activity coefficient of the NaBH_4_ electrolyte in water was calculated across a wide range of concentrations using the Pitzer model. To determine the solvation free energy of the borohydride ion, we employed the cluster-continuum method. Thermodynamic integrations were performed with varying LJ parameters σ_HB_ and ε_HB_ of the BH_4_^–^ hydrogen to calculate the solvation free energy. Our optimization approach ensures accurate reproduction of water–ion interactions and ion pairing, aligning with experimental findings. The resulting NaBH_4_ model enables accurate molecular dynamics simulations, capturing the relative distribution of ions in aqueous salt solutions, solvation free energies, and activities over a broad range of concentrations.

## Supporting Information Available

Experimental osmolality data, *ab initio* MD simulations, description of advanced solution structure analysis, comparison to naive force-field, MP2-optimized microhydrated structures and hydration free energies of anions, methodology used in force-field development, free energy of solvation, diffusion coefficient calculation, and refined parametrization of NaBH_4_ (PDF)
