# Supplementary material for: Unveiling the Borohydride Ion through Force-Field Development
Source: J Chem Theory Comput. 2024 Jan 16;20(3):1263–73. doi: 10.1021/acs.jctc.3c01020 (PMC10867804; doi:10.1021/acs.jctc.3c01020)
Supplement: Supplementary file 1 — ct3c01020_si_001.pdf [file ct3c01020_si_001.pdf]

Supporting Information – Unveiling the Borohydride Ion through Force-Field Development

Shavkat Mamatkulov,[†] Jakub Polák,[‡] Jamoliddin Razzokov,[¶,§] Lukáš Tomaník,[‡] Petr Slavíček,[‡] Joachim Dzubiella,[‖] Matej Kanduč,[⊥] and Jan Heyda[*,‡]

†Institute of material science of AS, Ch.Aytmatov str.2B, Tashkent, Uzbekistan

‡Department of Physical Chemistry, University of Chemistry and Technology, Prague, Technická 5, 16628 Prague 6, Czech Republic

¶Institute of Fundamental and Applied Research, National Research University TIIAME, Kori Niyoziy 39, Tashkent, 100000, Uzbekistan

§School of Engineering, Akfa University, Milliy Bog Street 264, Tashkent, 111221, Uzbekistan

‖Applied Theoretical Physics-Computational Physics, Physikalisches Institut, Albert-Ludwigs-Universität Freiburg, Hermann-Herder-Str. 3, D-79104 Freiburg, Germany

⊥Jožef Stefan Institute, Jamova 39, 1000 Ljubljana, Slovenia

E-mail: heydaj@vscht.cz

Pitzer model on NaCl/NaOH mixture and extrapolation to pH=7:

Experimental data

We conducted osmolality measurements on ternary NaCl/NaOH solutions. This enabled us to verify the reliability of an extrapolation scheme that employs known properties of ternary NaCl/NaOH mixtures to predict the properties of pure binary NaCl solutions. The raw experimental data are presented in Tab. S1, in which the water activities and osmotic coefficients are also evaluated (Eq. 1).

Table S1: Osmolality of ternary NaCl/NaOH mixed solutions as determined in VPO measurements at 310 K. The real ternary solutions, at finite NaOH concentrations, were employed during the fitting with the Pitzer model, while the binary NaCl solution was a target used to estimate the quality of the extrapolation.

$\underline{m}_{\mathrm{NaCl}}$	$\underline{m}_{\mathrm{NaOH}}$	Osmolality	$\ln a_{\mathrm{w}}$	ϕ
0.25	0	466.2	-0.00845	0.938817
0.5	0	888.8	-0.01611	0.894917
1.00	0	1892.4	-0.0343	0.952712
1.997	0	3867.2	-0.07009	0.974916
2.9626	0	6412.8	-0.11622	1.089742
6.0123	0	15178.2	-0.27509	1.270953
0	0.1	178.0	-0.00323	0.896125
1.9858	0.1	3831.7	-0.06945	0.92485
1.5046	0.1	2942.0	-0.05332	0.923037
0.4994	0.1	1077.7	-0.01953	0.90517
3.0146	0.1	5977.6	-0.10834	0.966214
5.9701	0.1	13388.8	-0.24266	1.110439
0.56578	0.4343	1800.6	-0.03263	0.906444
1.51701	0.49607	3524.8	-0.06388	0.8815
2.9261	0.5126	6867.8	-0.12447	1.005483
0.52973	0.99969	2599.7	-0.04712	0.85574
5.0158	0.92806	12097.3	-0.21925	1.024632
2.9737	1.1301	7782.4	-0.14105	0.95472

We have measured the osmolality of ternary solution of NaBH4/NaOH at $\underline{m}_{\mathrm{NaOH}} = 1\,\mathrm{mol/kg}$ (pH $\approx$ 14). The raw experimental data are presented in Tab. S2, in which the water activity

Table S2: Osmolality of ternary $NaBH_4$/NaOH mixed solutions as determined in VPO measurements at 310 K. The data served determining remaining (free) parameters in the Pitzer model.

$\underline{m}_{NaBH_4}$	$\underline{m}_{NaOH}$	Osmolality	$\ln a_w$	ϕ
1.1434	0.9999	4096	-0.07424	0.962112
1.18069	0.9999	4245.12	-0.07694	0.980087
2.0854	0.9999	6249.956	-0.11327	1.019831

and osmotic coefficient are also evaluated (Eqs. 1, 5). Experimental data for $\underline{m}_{NaOH} = 0.1$ mol/kg were ignored since our experiments proved that the heat associated with slow $NaBH_4$ decomposition at pH ≈ 13 affected our VPO signal (hundreds of mOsm units).

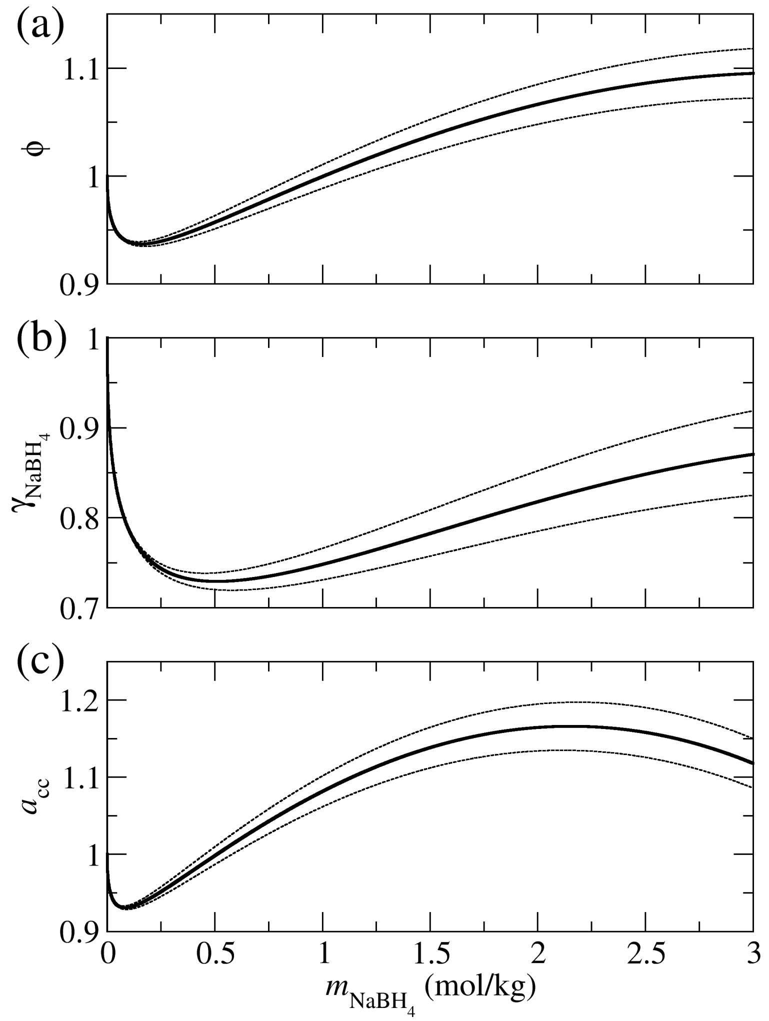

Figure S1: Non-ideality of $NaBH_4$ solution as quantified by (a) osmotic coefficient, (b) activity coefficient, and (c) activity coefficient derivative. Dashed lines represent the combined uncertainty arising from both experimental measurements and the fitting model.

MD Simulation: ab-initio MD disqualifies polar FF model of BH_4^-

Advanced analysis

In order to develop a reliable empirical force field for BH_4^-, we have analyzed in detail the anion hydration by ab initio MD. We highlight the differences between the results from ab-initio MD and a classical force-filed (FF) simulations, that would represent highly polar BH_4^- with $\Delta\Delta G_{\mathrm{solv}} \approx -78\,\mathrm{kJ/mol}$.

First, the radial distribution functions were determined for B–O_w, B–H_w, H–O_w, and H–H_w. Second, the directionality of the H–H_w interaction (i.e., H-H 'hydrogen' bond) was determined via the angle-resolved $g(r, \alpha)$. Last, the 3D-density maps of water (i.e., of water oxygen and water hydrogen) around BH_4^- were calculated and compared.

From ab initio simulations, we have also analyzed the distribution of bond lengths and angles between BH_4^- and the hydration water molecules, and, importantly, also the partial charges in BH_4^-. We observed a charge transfer from BH_4^- to the neighboring water molecules, which further lowers the polarity of BH_4^- and the partial charges of its hydrogen atoms.

The radial distribution function $g(r)$ was calculated and normalized as usual. The spatial distribution function $g(\vec{r})$ is defined via Eq. 1, i.e., as a ratio of a local and a bulk density at a position $\vec{r}_{\mathrm{B}i}$. We note that the central particle is the boron atom (placed at [0,0,0]), with the best possible orientation of two hydrogen atoms, where the first one defines x-axis. With the second hydrogen, they define the reference xyz-coordinate system. In order to symmetrize and smoothen the 3D-spatial distribution function, we have averaged over all possible hydrogen atom permutations. We have calculated the spatial distribution of water oxygen and hydrogen with the spatial resolution of $a_{\mathrm{bin}} = 0.25\,\mathrm{\AA}$, i.e., volume element $V = 0.0156\,\mathrm{\AA}^3$.

$$g(\vec{r}_{\mathrm{B}i}) = \frac{\rho(\vec{r}_{\mathrm{B}i})}{\rho_i^{\mathrm{bulk}}} \tag{1}$$

$$\rho(\vec{r}_{\mathrm{B}i}) = \frac{\langle N_i(\vec{r}_{\mathrm{B}i})\rangle}{V_{\mathrm{bin}}(\vec{r}_{\mathrm{B}i})} \tag{2}$$

In order to calculate the angle-resolved radial distribution function for water hydrogens, we proceeded in two steps (see Figure 5). First, the distance between boron and water hydrogen was calculated ($r_{\mathrm{BH_w}}$). Then the closest boron hydrogen to water hydrogen was found ($r_{\mathrm{HH_w}}$) and the angle $\mathrm{H_{nearest}}$–B–$\mathrm{H_w}$ was calculated, see the sketch in Figure 5. The normalization is performed with respect to both, the angular (α) and spatial coordinate (r-distance).

Results

In this section, we compare the hydration structure of BH_4^- anion obtained from ab-initio MD simulation and force-field that represents polar BH_4^- anion that reproduces the literature estimate of $\Delta\Delta G_{\mathrm{solv}} \approx -78\,\mathrm{kJ/mol}$.

The radial distribution functions, presented in Figure S2, obtained from ab-initio and from force-field simulations are clearly different. Most importantly, at the ab-initio level the hydration water around BH_4^- is relatively dispersed, and the $g(r)$ do not exhibit pronounced structure. Therefore, in ab-initio simulations, BH_4^- behaves similar to a large anion with a small charge density. In contrast, highly polar empirical force field exhibits sharp peaks in the first and second hydration shell, thus signaling very specific hydration of BH_4^- anion. This difference could be roughly related to the signatures of typical force-field hydration of NH_4^+ vs. CH_4. In the former case, the solute–water H-bonding features are important. In the latter, the water H-bond network from bulk water is essentially preserved, just encapsulating the 'large' spherical solute (hydration of small hydrophobic solutes).

Comparing the 2D distribution functions in Figure S3, further clear differences between ab-initio and FF simulations are observed. First, the empirical force field exhibits much stronger orientational preferences (see yellow spots at low-α, $r < 4\,\mathrm{Å}$). Secondly, the B–$\mathrm{H_w}$ (and consequently also H–$\mathrm{H_w}$) distance is shorter by about $0.1\,\mathrm{Å}$ compared to the ab-initio result. See also the discussion related to the RDFs presented in Figure S2.

The strong orientation of water in MD FF and directionality of the H–$\mathrm{H_w}$ bonds, which

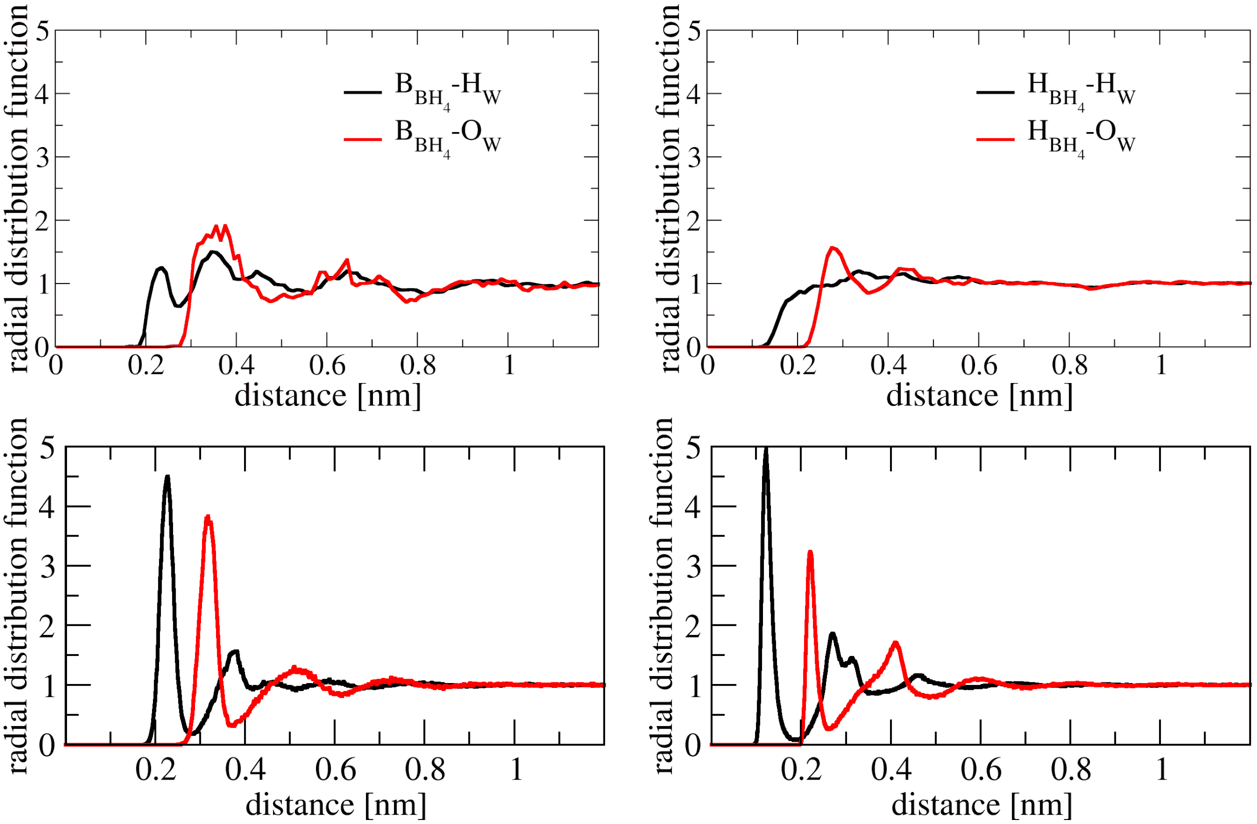

Figure S2: Radial distribution function between boron and water (left, B-H_w black, B-O_w red) and hydrogen atoms of BH_4^- and water (right, B-H_w black, B-O_w red). Hydration structures obtained from ab-initio MD simulations of a spherical domain (radius 7.5 Å described at QM level) embedded in TIP3P force-field water (top) and an empirical force-field (bottom, polar BH_4^- of $\Delta\Delta G_{\mathrm{solv}} \approx -78\,\mathrm{kJ/mol}$) MD simulation are compared.

are found in 1D and 2D analysis (i.e., in Figures S2 and S3), are also apparent from random snapshots taken from both types of simulations in Figure S4. We can see that the orientation of water molecules around the BH_4^- anion remains water-like in the case of ab-initio simulation (left), while it is highly ordered in the case of force-field simulation (right).

The intrinsic geometries of the hydration layer are quantified by the spatial distribution function in Figure S5. Here the relative disorder and low magnitude of the local density ($\approx 2\times$ or $3\times$ the bulk) vs. directionality and high magnitude of the local density ($\approx 15\times$ the bulk) are well documented in the case of ab-initio MD (top) and FF MD (bottom), respectively.

Table S3 presents the significantly different charge distribution within the BH_4^- anion pre-

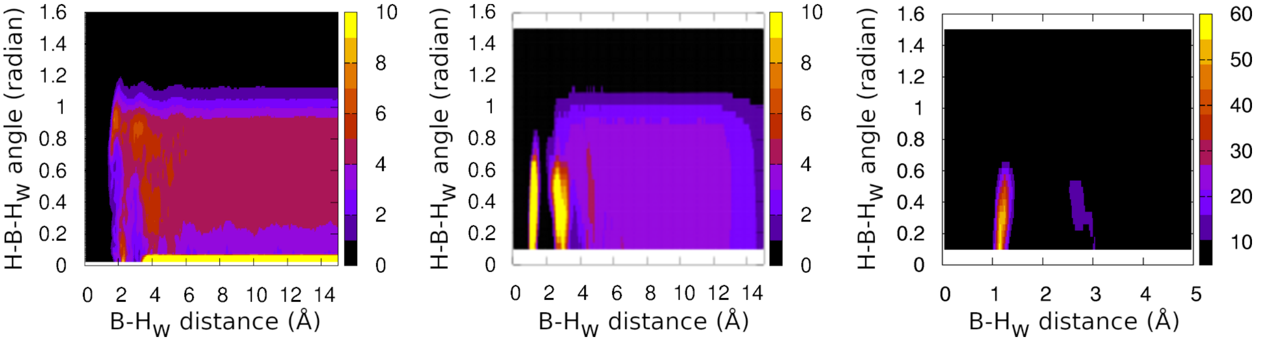

Figure S3: Probability distribution of H_w–$H_{BH_4^-}$ orientations, defined by the α-angle, as explained in Figure 8 in the main text. The distance from a water hydrogen atom to the central boron atom is measured, and the angle is defined between the closest boron hydrogen, the boron atom, and the water hydrogen. The 2D-distribution obtained from ab-initio MD simulation shown is on the left, while the FF-based result ($\Delta\Delta G_{\mathrm{solv}} \approx -78\,\mathrm{kJ/mol}$) is presented in the middle. Note that the visualization has been capped at a maximum probability of 10 for clarity, but the full peak height is displayed on the right.

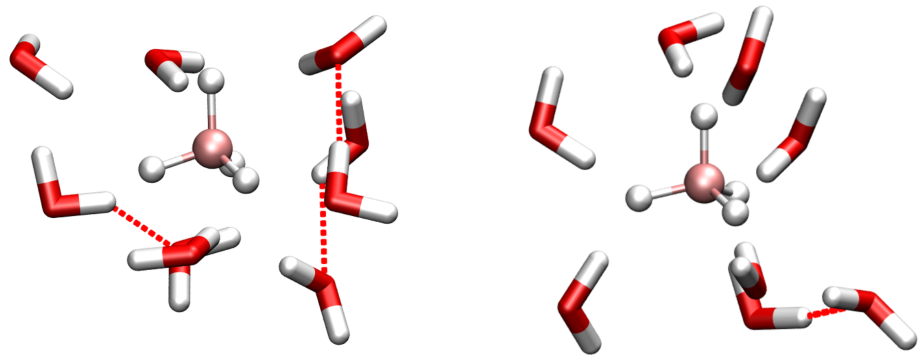

Figure S4: Geometry of the first hydration layer of the BH_4^- anion obtained from ab-initio (left) and force-field (right, $\Delta\Delta G_{\mathrm{solv}} \approx -78\,\mathrm{kJ/mol}$) simulations. Note the significantly enhanced directionality of water–anion H–H_w bonding in the case of the force field simulation, and bulk-like H-bonding structure in the case of the ab-initio simulation.

dicted by ab-initio (MD) with charge transfer ($q_{\mathrm{B}} = -0.56e$, $q_{\mathrm{H}} = -0.11e$), which contrasts with ($q_{\mathrm{B}} = +1.2e$, $q_{\mathrm{H}} = -0.55e$) used in the FF simulation to match $\Delta\Delta G_{\mathrm{solv}} \approx -78\,\mathrm{kJ/mol}$ (literature estimate).

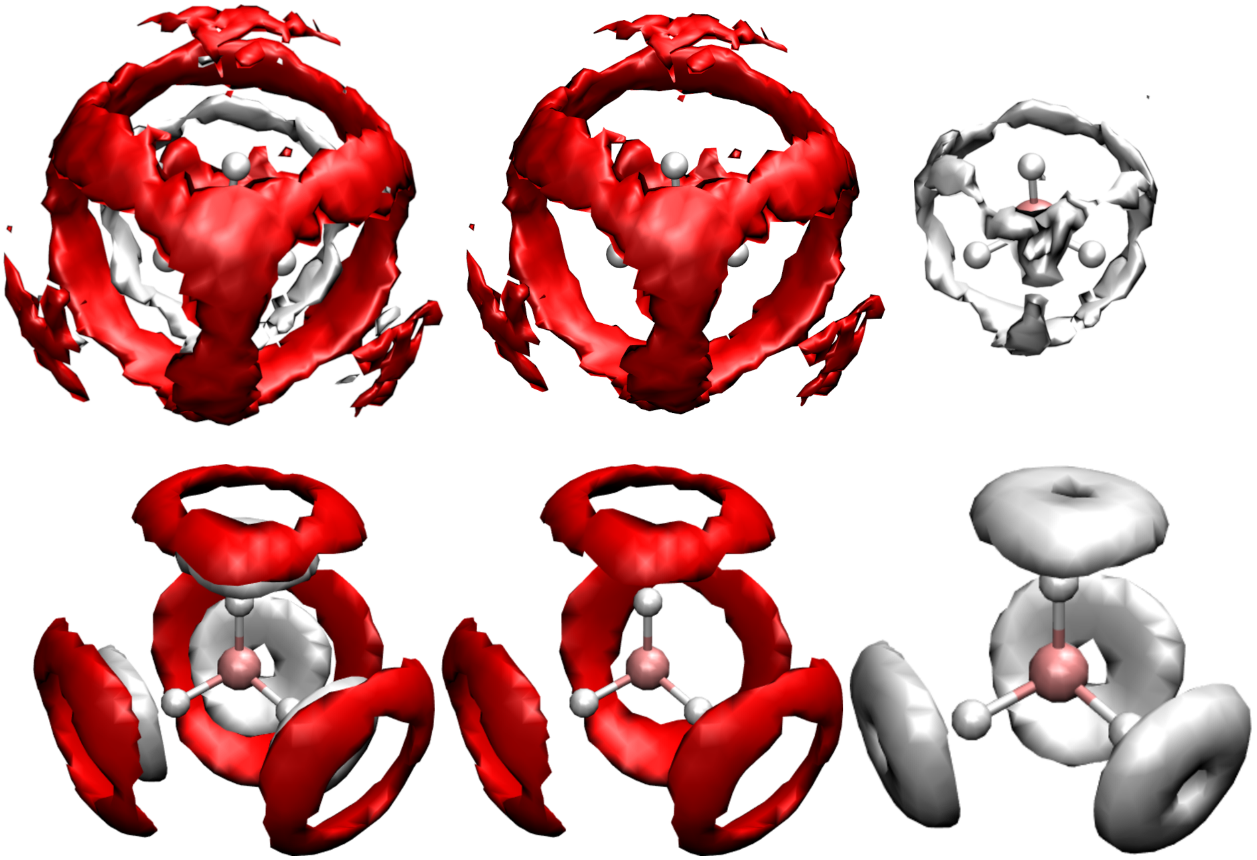

Figure S5: Spatial distribution functions of water hydrogen (white) and oxygen (red) atoms around the BH_4^- anion. The ab-initio results are shown on the top, and the force field simulation results (polar BH_4^- of $\Delta\Delta G_{\mathrm{solv}} \approx -78\,\mathrm{kJ/mol}$) at the bottom. The isocontour level of the spatial distribution is enhanced by 3× for O_w and 2× for H_w relative to the bulk density in the case of ab-initio MD, and 15× (both O_w, H_w) in the case of FF simulations.

Table S3: Partial charges on the neutral borane (BH_3) and borohydride anion (BH_4^-) as calculated at different levels of theory. The second column presents the results of gas-phase calculation, the third column presents the result from ab-initio MD of BH_4^- in a spherical water cluster. In the case of gas-phase, the partial charges are derived using the RESP method. In ab-initio MD simulation, the unrestricted (EPS) partial charges were calculated. Moreover, it was found that about 20 % of the charge (i.e., $0.2\,e$) was transferred to the water molecules in the first hydration layer, therefore also the rescaled values of the partial charges (total charge of BH_4^- $q = -1e$) are also shown (fourth column). The partial charges employed in the empirical FF of highly polar BH_4^- are shown in the last column for comparison.

Atom	BH_3 (GP)	BH_4^- (ab-init.MD)	BH_4^- (rescaled)	BH_4^- (FF, $\Delta\Delta G_{\mathrm{solv}} \approx -78\,\mathrm{kJ/mol}$)
B	0.525	−0.4	−0.56	+1.2
H	−0.175	−0.08	−0.11	−0.55

QM: Hydration Free Energy

Hydration free energy from cluster continuum approach

Table S4: Cluster–continuum approach (CCA) to calculate ΔG_{solv} for different numbers ($N_{\mathrm{wat}} = 0-3$) of explicitly involved water molecules (solvation energies in kJ·mol^{-1}) calculated at MP2/aug-cc-pVTZ employing PCM with the Bondi radii.

N_{wat}	Cl^-	BH_4^-	BF_4^-
**0**	−305	−274	−254
**1**	−307	−263	−234
**2**	−298	−248	−224
**3**	−299	−261	−231

Microhydrated structures of anions

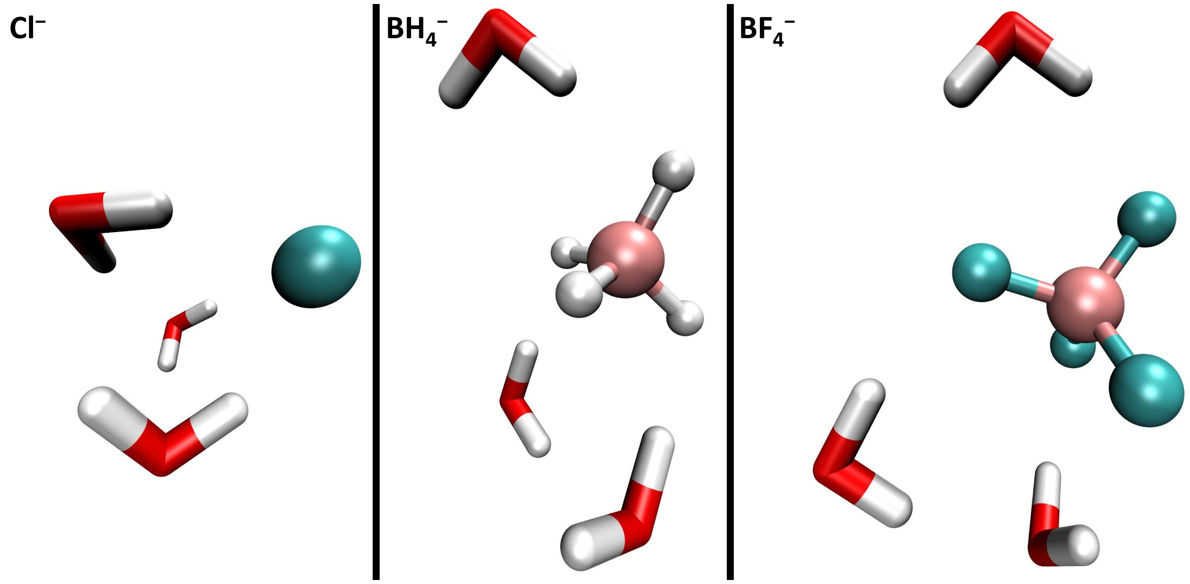

Figure S6: Microhydrated structures of Cl^-, BH_4^-, and BF_4^- optimized on the MP2/aug-cc-pVTZ level of theory. While ordered and strongly directional interactions between water molecules and Cl^- anion are observed, less structured hydration is observed for BH_4^- and BF_4^-.

Methodology – Force Field Development – Classical MD

Ab initio computations

We have performed ab initio computations to optimize the structure of the borohydride ion in the gas phase and in water using the MP2/6311++G(p,d) basis set. The single point polarised continuum model (PCM) was used to take the long-range electrostatic effect of solvent into account. All calculations are carried out with the GAUSSIAN 16 program package.[1]

The borohydride ion consists of a central boron atom surrounded by four hydrogen atoms in a tetrahedral arrangement. The equilibrium geometry of BH_4^- in water has the H–B–H angle of 109.5^0 and the B–H bond length of 0.124 nm. The partial charges were generated using the CHELPG tool of the GAUSSIAN program. The values of the partial atomic charges of $q_{\mathrm{B}} = 0.134\,e$, $q_{\mathrm{H}} = -0.2835\,e$ were obtained and they are close to the results of the work of Fang et al.[2]

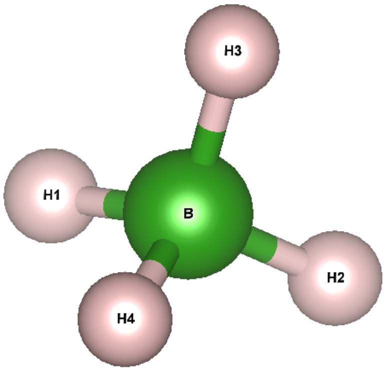

Figure S7: Borohydride ion with a labeling notation of individual atoms.

Molecular dynamics (MD) simulations

The pair interaction potential between atoms V_{ij} is modeled through the sum of Coulomb and Lennard-Jones (LJ) interactions

$$V_{ij}(r_{ij}) = \frac{1}{4\pi\varepsilon_0}\frac{q_i q_j}{r_{ij}} + 4\varepsilon_{ij}\left[\frac{\sigma_{ij}^{12}}{r_{ij}^{12}} - \frac{\sigma_{ij}^{12}}{r_{ij}^{6}}\right], \tag{3}$$

where q_i, q_j are the charges of atoms i, j and r_{ij} is the distance between these atoms.

We use the Lorentz–Berthelot combination rules to define the Lennard-Jones parameters

$$\varepsilon_{ij} = \sqrt{\varepsilon_i \varepsilon_j}, \quad \sigma_{ij} = \frac{\sigma_i + \sigma_j}{2} \tag{4}$$

where i, j correspond to the index of the atoms and ions.

We employ the SPC/E water model.[3] The model assigns partial charges of −0.8476 and 0.4238 to oxygen and hydrogen, respectively. The water geometry is fixed at a bond length of 1.0 Å and a bond angle of 109.47° using the LINCS algorithm.[4] For Na^+ and Cl^-, we use the Smith-Dang parameters.[5]

Free energy of solvation

The solvation free energy of the BH_4^- ion is calculated using thermodynamic integration[6]

$$\Delta G_{\mathrm{sim}} = \int_0^1 \left\langle \frac{\partial H_\lambda(\lambda_{\mathrm{LJ}}, \lambda_{\mathrm{C}} = 0)}{\partial \lambda_{\mathrm{LJ}}} \right\rangle \mathrm{d}\lambda_{\mathrm{LJ}} + \int_0^1 \left\langle \frac{\partial H_\lambda(\lambda_{\mathrm{LJ}} = 1, \lambda_{\mathrm{C}})}{\partial \lambda_{\mathrm{C}}} \right\rangle \mathrm{d}\lambda_{\mathrm{C}} ,$$

where H_λ is the Hamiltonian of the system, λ_{LJ} and λ_{C} are the LJ and charge transition coordinates, which are 0 in the initial state and 1 in the final state. The solvation path is split in two separate processes: first, a neutral van der Waals particle is created, which is assigned a charge in the second step. Along the transition path, the λ-dependent Hamiltonian is

defined as

$$H_\lambda(\lambda_{\rm LJ}, \lambda_{\rm C}) = H\{q_{\rm ion} = q\lambda_{\rm C},\, \sigma_{\rm ion} = \sigma[1-(1-\lambda_{\rm LJ})^k],\, \varepsilon_{\rm ion} = \varepsilon[1-(1-\lambda_{\rm LJ})^k]\}.$$

We set the exponent $k = 6$ in this equation to avoid divergences. Integrations are performed through a 12-point Gaussian quadrature with $\lambda \in \{0.00922, 0.04794, 0.11505, 0.20634, 0.31608, 0.43738, 0.56262, 0.68392, 0.79366, 0.88495, 0.95206, 0.99078\}$. For every value of λ, we perform a 400 ps simulation of which the first 50 ps are discarded for equilibration.

The ionic solvation free energy computed in the simulations is sensitive to the simulation scheme (system shape, periodic or finite system) and treatment of the electrostatic forces (Ewald sum, cut-off based, etc). Therefore for comparison with experimental data, several corrections have to be applied to the simulation data. The correction term accounting for finite system and ion size reads[7]

$$\Delta G_{\rm fs} = \frac{z^2 N_{\rm A} e^2}{4\pi\varepsilon_0^2}\Big[-\frac{\xi_{\rm ew}}{2\varepsilon_{\rm r}} + \Big(1+\frac{1}{\varepsilon_{\rm r}}\Big)\Big(\frac{2\pi R_{\rm ion}^2}{3L^3} - \frac{4\pi^2 R_{\rm ion}^5}{45L^6}\Big)\Big]$$

where z is the ion valency and e the elementary charge. Here, $R_{\rm ion}$ is the effective radius of the ion, which is estimated from the ion–oxygen radial distribution function, and $\varepsilon_{\rm r}$ is the relative dielectric constant of the SPC/E water. The Wigner potential is $\xi_{\rm ew} = -2.837279/L$, where L is the simulation box size in nm.

Experimental values of the solvation free energy are usually given with respect to a hypothetical transfer of ions from the ideal gas phase of $p_0 = 1$ atm pressure to the ideal solution under pressure of $p_1 = 24.6$ atm, corresponding to a density of 1 mol/l. Thus, it is also necessary to include a correction term related to the compression of the gas

$$\Delta G_{\rm press} = N_{\rm A} k_{\rm B} T \ln(p_1/p_0) = 7.9 \text{ kJ/mol}$$

where $N_{\rm A}$ and $k_{\rm B}T$ are Avogadro's number and the thermal energy, respectively.

Hence, the total single-ion solvation free energy is given by

$$\Delta G_{\text{solv}} = \Delta G_{\text{sim}} + \Delta G_{\text{fs}} + \Delta G_{\text{press}}. \tag{5}$$

Solvation free energy and diffusion coefficient

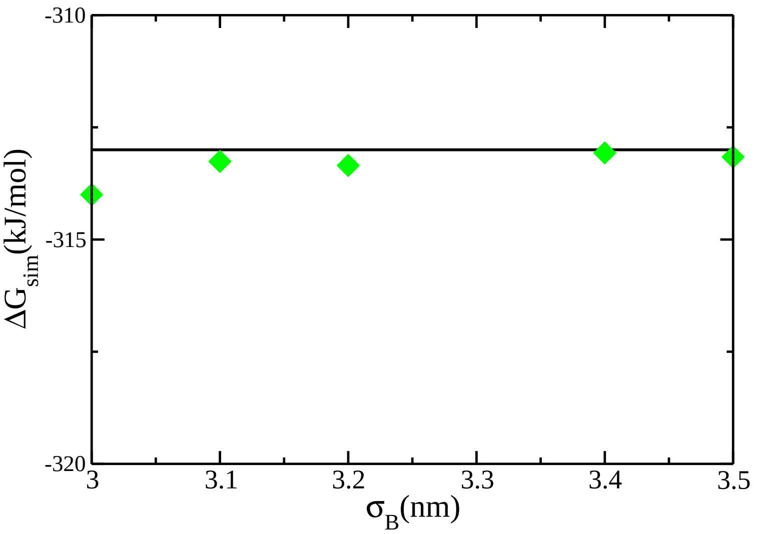

Figure S8: The solvation free energy of BH_4^- ion as a function of boron atom radius. The green symbols show the MD simulation results for solvation free energy. The partial charges and LJ interaction parameters of the borohydride ion are $q_{\text{B}} = 0.108\,e$, $q_{\text{HB}} = -0.277\,e$, $\varepsilon_{\text{B}} = 0.4\,\text{kJ/mol}$, $\sigma_{\text{HB}} = 0.34\,\text{nm}$, and $\varepsilon_{\text{HB}} = 0.5\,\text{kJ/mol}$. As boron atoms are screened by hydrogen atoms, changing their radius does not affect the solvation free energy.

Self-diffusion coefficient of a single ion in water:

For the final LJ parameters of BH_4^-, the self-diffusion coefficient is calculated from an additional 100 ns NVT ensemble simulation of the single ions in different cubic box sizes ($L =$ 3.0, 4.0, and 5.0 nm). All simulations are pre-equilibrated in the NPT ensemble before fixing the box size. The self-diffusion coefficient of the borohydride ion is calculated from the slope of the mean-square displacement by a linear fit as shown in Fig.S9. The diffusion coefficient

corrected for system size effects is calculated using the formula:[8]

$$D_0 = D_{\text{pbc}}(L) + \frac{k_B T \xi_{\text{ew}}}{6\pi\eta L} \tag{6}$$

where L is the box length, D_{pbc} the computed self-diffusion coefficient, D_0 the diffusion coefficient for infinite non-periodic systems, k_B the Boltzmann constant, T the absolute temperature, η the solvent viscosity, and $\xi_{\text{ew}} = 2.837297$ the self-term for the cubic lattice. To correct for the low viscosity of the SPC/E water compared to the measured water viscosity, we report the scaled diffusion coefficients,[9]

$$D = \frac{\eta_{\text{spce}}}{\eta_{\text{water}}} D_0 \tag{7}$$

with the viscosity of SPC/E water $\eta_{\text{spce}} = 7.29 \times 10^{-4}\,\text{kg m}^{-1}\text{s}^{-1}$ and the experimentally measured water viscosity $\eta_{\text{water}} = 8.91 \times 10^{-4}\,\text{kg m}^{-1}\text{s}^{-1}$.[10] The diffusion coefficient value of the borohydride ion at 300 K is $D = 1.96 \times 10^{-5}\,\text{cm}^2\ \text{s}^{-1}$ and agrees well with the experimentally reported value of $D = 2.01 \times 10^{-5}\,\text{cm}^2\ \text{s}^{-1}$.[11]

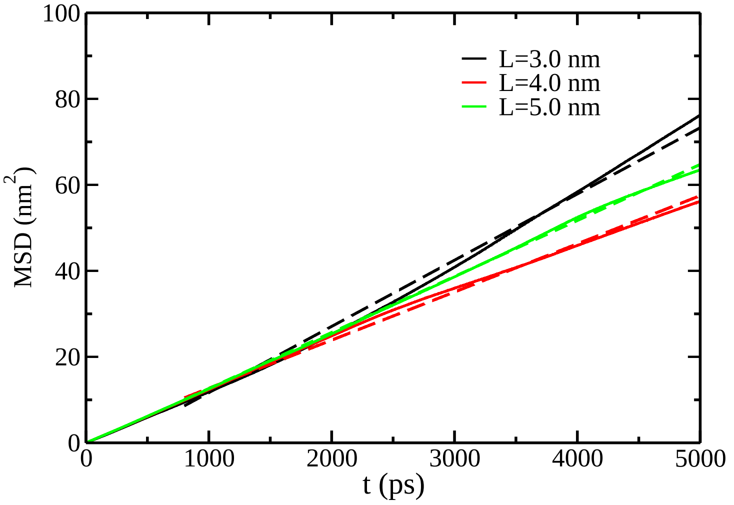

Figure S9: Mean square displacement BH_4^- ion using the SPC/E water model. Different color lines refer to different box sizes. Diffusion coefficients are obtained by a linear fit from 0.8 to 5 ns shown as dotted lines.

Borohydride ion topology

Include forcefield parameters

[defaults]

nbfunc comb-rule gen-pairs fudgeLJ fudgeQQ

1 2 no 1.0 0.833

[atomtypes]

name at.num mass charge ptype sigma epsilon

B 16 10.8060 0.144 A 3.48e-01 0.4

HM 1 1.0080 -0.286 A 3.4e-01 0.570

DB 16 10.8060 0.000 A 0.00000e+01 0.0e+00

DH 1 1.0080 0.000 A 0.00000e+00 0.0e+00

OW 8 15.9994 0.000 A 3.16571e-01 6.5000e-01

HW 1 1.0080 0.000 A 0.00000e+00 0.0000e+00

Na+ 11 22.98980 1.000 A 2.58300e-1 0.4186

[moleculetype]

Name nrexcl

BH4 3

[atoms]

nr type resnr residue atom cgnr charge mass

1 B 1 BH4 B 1 0.108 10.8060 B 0.0 10.086

2 HM 1 BH4 H1 1 -0.277 1.0080 HM 0.0 1.0080

3 HM 1 BH4 H2 1 -0.277 1.0080 HM 0.0 1.0080

4 HM 1 BH4 H3 1 -0.277 1.0080 HM 0.0 1.0080

5 HM 1 BH4 H4 1 -0.277 1.0080 HM 0.0 1.0080

[bonds]

ai aj funct c0 c1 c2 c3

1 2 1 0.12400 376560.

1 3 1 0.12400 376560.

1 4 1 0.12400 376560.

1 5 1 0.12400 376560.

[angles]

ai aj ak funct c0 c1 c2 c3

2 1 3 1 109.500 520.000

2 1 4 1 109.500 520.000

2 1 5 1 109.500 520.000

3 1 4 1 109.500 520.000

3 1 5 1 109.500 520.000

4 1 5 1 109.500 520.000

#include ”spce.itp”

[moleculetype]

molname nrexcl

Na+ 1

[atoms]

id at type res nr residu name at name cg nr charge mass

1 Na+ 1 Na+ Na 1 1 24.30500

[system]

Name

Boronhydrate

[molecules]

Compound mols

BH4 200

SOL 4916

Na+ 200

References

(1) Frisch, M. et al. Gaussian 09 Revision, D.01. Gaussian Inc. Wallingford CT 2009.

(2) Zhou, Y.; Yoshida, K.; Yamaguchi, T.; Liu, H.; Fang, C.; Fang, Y. Microhydration of BH_4^- : Dihydrogen Bonds, Structure, Stability, and Raman Spectra. *The Journal of Physical Chemistry A* **2017**, *121*, 9146–9155.

(3) Berendsen, H. J. C.; Grigera, J. R.; Straatsma, T. P. The missing term in effective pair potentials. *The Journal of Physical Chemistry* **1987**, *91*, 6269–6271.

(4) Ryckaert, J.-P.; Ciccotti, G.; Berendsen, H. J. Numerical integration of the cartesian equations of motion of a system with constraints: molecular dynamics of n-alkanes. *Journal of Computational Physics* **1977**, *23*, 327–341.

(5) Dang, L. X.; Smith, D. E. Molecular dynamics simulations of aqueous ionic clusters using polarizable water. *The Journal of Chemical Physics* **1993**, *99*, 6950–6956.

(6) Straatsma, T. P.; McCammon, J. A. Computational Alchemy. *Annual Review of Physical Chemistry* **1992**, *43*, 407–435.

(7) Zhan, C.-G.; Dixon, D. A. First-Principles Determination of the Absolute Hydration Free Energy of the Hydroxide Ion. *The Journal of Physical Chemistry A* **2002**, *106*, 9737–9744.

(8) Yeh, I. C.; Hummer, G. System-size dependence of diffusion coefficients and viscosities from molecular dynamics simulations with periodic boundary conditions. *Journal of Physical Chemistry B* **2004**, *108*, 15873–15879.

(9) Mamatkulov, S.; Schwierz, N. Force fields for monovalent and divalent metal cations in TIP3P water based on thermodynamic and kinetic properties. *The Journal of Chemical Physics* **2018**, *148*, 074504.

(10) Vega, C.; Abascal, J. L. F. Simulating water with rigid non-polarizable models: a general perspective. *Physical Chemistry Chemical Physics* **2011**, *13*, 19663–19688.

(11) Wang, K.; Lu, J.; Zhuang, L. Direct determination of diffusion coefficient for borohydride anions in alkaline solutions using chronoamperometry with spherical Au electrodes. *Journal of Electroanalytical Chemistry* **2005**, *585*, 191–196.
